# ‘Cough and sneeze into your elbow’: a field study testing the effects of persuasive messages on compliance with behavioral measures to prevent the spread of respiratory viruses

**DOI:** 10.1080/21642850.2026.2616931

**Published:** 2026-01-20

**Authors:** Amy van der Heijden, Anne Vos, John de Wit, Daniëlle Timmermans, Bas van den Putte

**Affiliations:** a Amsterdam School of Communication Research (ASCoR), University of Amsterdam, Amsterdam, The Netherlands; b Department of Health Sciences, Vrije Universiteit Amsterdam, Amsterdam, The Netherlands; c Department of Interdisciplinary Social Science, Utrecht University, Utrecht, The Netherlands; d Amsterdam University Medical Centers, Amsterdam, The Netherlands

**Keywords:** Persuasive communication, health communication, respiratory infections, COVID-19, pandemic preparedness

## Abstract

**Background:**

Effective persuasive messages can contribute to enhancing pandemic preparedness and public health. An essential requirement for this is an excellent understanding of the effects of exposure to persuasive messages on compliance with behavioral measures against the spread of respiratory viruses. This field study tested the effects of persuasive messages on compliance with two behavioral measures to prevent the spread of viruses that cause respiratory infections such as COVID-19 and the flu: coughing/sneezing into the elbow and staying home when ill with respiratory infection symptoms.

**Materials and methods:**

A field study with an observational pre-post design was conducted at four educational institutions representing all common post-secondary school educational levels in the Netherlands. Data were collected among students and employees via online questionnaires before (*n* = 2096) and after (*n* = 1098) exposure to a set of persuasive messages with six different message framings. Two-way MANOVA, logistic regression analysis and repeated measures ANOVA were conducted.

**Results:**

Exposure frequency, behavioral measure type, demographic characteristics, trust in government, prosocial orientation, perceived health, educational institution, and student/employee status showed significant multivariate main effects and univariate main and/or interaction effects on the outcomes (intention to comply, attitude, social norms, moral norm, self-efficacy, response-efficacy, and risk perception; *p* < .05). The odds of compliance with staying home when ill were lower than the odds of compliance with coughing/sneezing into the elbow, regardless of exposure frequency (*p* < .001).

**Conclusion:**

In conclusion, messages positively influenced behavioral determinants – a critical prerequisite for behavior change and compliance. Findings also highlight that people are likely more willing to comply with measures that have less adverse personal and social impact. To enhance compliance more is needed, for instance explanations of the relevance and effectiveness of measures, and practical support to enact the measures. Practical and theoretical implications are discussed.

## Introduction

The COVID-19 pandemic had major impact on societies worldwide. The World Health Organization (WHO) reported that 776 million people globally became infected with the coronavirus since the start of the outbreak in late 2019, resulting in the death of over 7 million people. In Europe, the cumulative number of infections and deaths is 280 million and over 2 million, respectively (WHO, 2024). The pandemic put a lot of pressure on healthcare systems (Trentini et al., 2022; Van Giessen et al., 2020), posed a large economic burden (Faramarzi et al., 2024; Richards et al., 2022), and had major impact on individuals' mental health (COVID-19 Mental Disorders Collaborators, 2021).

Behavioral and environmental measures were implemented by governments across countries in attempts to curb the number of infections, including physical distancing, wearing face masks, regular and appropriate hand washing, self-isolation, and closing public institutions and buildings such as schools and work places (e.g. Talic et al., 2021). It is shown that combinations of implemented measures in the Netherlands were effective in reducing the number of infections, while acknowledging that the effectiveness of each individual measure is difficult to specify, and while also concluding that people's compliance with behavioral measures is essential for their effectiveness (Wallinga et al., 2024).

Prior research investigated effects of various types of communication strategies, such as different ways of framing, on behavioral determinants and compliance with COVID-19 preventive measures (e.g. Gillman et al., 2022; Hong & Hashimoto, 2021; Jiang & Amponsah Dodoo, 2021). A recent literature review showed that communication can have a major impact on compliance, but that several strategies had mixed effects (Williams et al., 2023). The review also noted that communication is itself a multi-faceted construct which makes it difficult to separate the impact of any one communication strategy, and to distinguish the effects of communication from voluntary and enforced behavioral recommendations and changes in the physical environment (Williams et al., 2023).

Prior research also explored effects of repeated exposure to information on behavioral determinants. Exposure to messages more frequently than desirable can lead to message fatigue, i.e. exhaustion and boredom after prolonged and repeated exposure to similar messages over time (So et al., 2016). While exposure to messages is essential to induce changes in determinants and behavior (Hornik, 2002), the relationship between message exposure and behavioral determinants is shown to take an inverted U-shape (So et al., 2016). More frequent exposure first leads to an increase, and then a decrease in behavioral determinants such as intention to perform specific behaviors. The optimal exposure length and frequency are still opaque. Meanwhile, health communication studies have shown that message fatigue may indeed induce resistance to promoted behavioral measures such as COVID-19 vaccine uptake (Cho & Salmon, 2007; Okuhara et al., 2023; Shen et al., 2022; Skurka & Keating, 2024; Zhao et al., 2023).

Although prior studies provided essential insights into the effects of various communication strategies, they were typically conducted in controlled environments such as online settings or labs. Evidence on effects of persuasive messages on compliance with measures to prevent respiratory virus spread in real-life practice is scarce, yet indispensable to enhance pandemic preparedness. Some field studies have been conducted, and these provide indications regarding if and how persuasive communication can enhance compliance in real life. For instance, a field study on a university campus in the Netherlands showed that interventions arousing empathy may enhance physical distancing (De Ridder et al., 2021). Another field study in the Netherlands showed that nudging and boosting effectively improved hand hygiene compliance in hospital nurses (Van Roekel et al., 2021). Although these prior studies are informative, the evidence base remains scarce. This is especially the case considering that each prior study deployed different intervention and communication strategies, targeting different behaviors among different study populations and in different contexts. A comprehensive understanding of the effects of exposure to persuasive messages on compliance with measures to prevent the spread of respiratory viruses in real-life is lacking, yet essential to effectively enhance pandemic preparedness.

### This study

The present study addresses this critical knowledge gap by exploring the effects of exposure to persuasive messages in real-life educational settings on (behavioral determinants of) compliance with two behavioral measures to prevent the spread of viruses that cause respiratory infections, notably: coughing and sneezing into the elbow, and staying home when ill with respiratory infection symptoms. These measures continue to be advised by the Dutch government in the current low-risk pandemic context to prevent the spread of a range of respiratory viruses, including COVID-19 and the flu (Government of the Netherlands, n.d.). Compliance and behavioral determinants were measured before and after exposure to persuasive messages about the two measures. Compliance with these behavioral measures following exposure to persuasive messages in a hypothetical high-risk pandemic situation was also explored.

The following hypotheses and exploratory research questions are addressed:

**H1.** Participants who are more frequently exposed to persuasive messages have a higher intention to comply with behavioral measures than participants who are less frequent or not exposed.

**H2.** Participants who are more frequently exposed to persuasive messages comply with behavioral measures more often than participants who are less frequent or not exposed.

**RQ1.** To what extent does the effect of more frequent exposure to persuasive messages (vs. less frequent or no exposure) on intention to comply with behavioral measures and other behavioral determinants differ as a function of:

- behavioral measure (coughing and sneezing into the elbow, or staying home when ill with respiratory infection symptoms)?
- educational institution (research university, university of applied sciences, intermediate vocational education), and role (student or employee)?
- demographic characteristics: age, gender, and ethnic background?
- personal characteristics: trust in government, prosocial orientation, perceived health status, and knowledge of respiratory viruses and preventive measures?

**RQ2.** To what extent do changes in intention and other behavioral determinants between pre- and post-exposure measurement differ as a function of exposure frequency?

**RQ3.** To what extent does participants' intention to comply with behavioral measures following exposure to persuasive messages in a hypothetical high-risk pandemic situation, differ from their intention to comply in the current low-risk situation?

## Materials and methods

### Study design, procedures and participant eligibility

**Figure 1.**
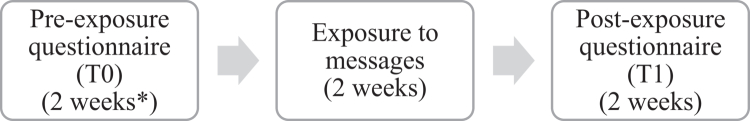
Field study design. Note: *The time frame for filling in the T0 and T1 questionnaires at the intermediate vocational education institution was one rather than two weeks, due to limited time to conduct the study at this institution.

A field study with an observational pre-post design was conducted at two research universities, a university of applied sciences, and an intermediate vocational education institution in the Netherlands. Data were collected from students and employees in November–December 2023 via self-administered online questionnaires before (pre-exposure, T0) and after (post-exposure, T1) exposure to persuasive messages about coughing and sneezing into the elbow, and staying home when ill with respiratory infection symptoms (Figure 1). Messages were disseminated simultaneously in Dutch and English, digitally and physically, via participating educational institutions' newsletters, websites, apps for students and staff, and in educational buildings via narrowcasting screens, billboards in elevators, and bulletin boards. Links to the questionnaires were distributed via the same channels, and by on-site by research assistants who asked students and employees in the educational buildings to participate via a QR-code. Participants at the research universities and the university of applied sciences were randomly allocated to answer questions related to either of the two behavioral measures. Participants at the intermediate vocational education institution only received questions about coughing and sneezing into the elbow, as they were also only exposed to messages about this measure, per request of the educational institution.

Participants were eligible when they were ≥16 years of age and affiliated as either student or employee with one of the participating educational institutions. No other exclusion criteria were applied. Participants received either study credits, entry to a gift voucher lottery, or a snack, depending on institution and role. Students and employees were encouraged to participate in both the pre- and post-exposure surveys, but could also complete only one (or none). Therefore, participants in T1 included individuals who completed both T0 and T1, as well as those who only participated in T1. A sample size calculation using G*Power (Faul et al., 2009) indicated that 1000 participants per measurement point were required to detect a small effect size of .10 at the standard .05 alpha error probability and a power of ß = .80.

Informed consent was obtained digitally on the first page of the questionnaire, which provided detailed study information and contact details of the researchers. The study was pre-registered at the Open Science Framework (Van der Heijden et al., 2023b), and approved by the Faculty Ethics Review Board of the Faculty of Social and Behavioral Sciences, University of Amsterdam (reference number FMG-4416).

### Materials: persuasive messages

**Figure 2.**
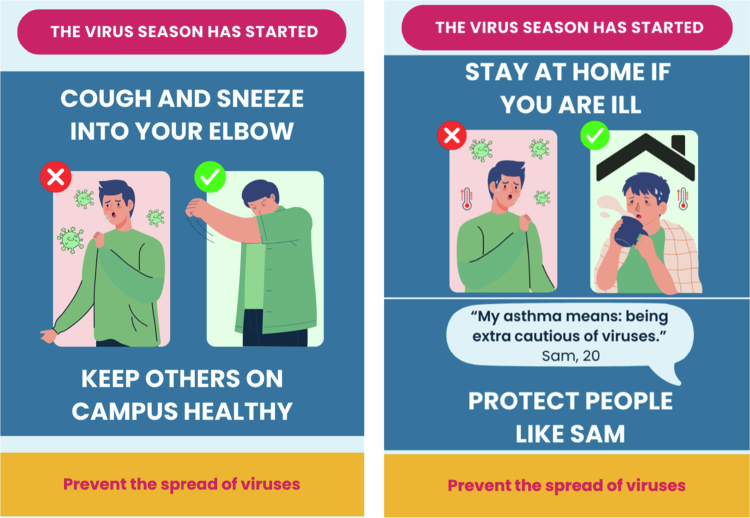
Examples of the posters conveying persuasive messages.

Six persuasive messages were designed in a poster format (Figure 2 and Supplementary File 1). Three messages related to coughing/sneezing into the elbow, and three to staying home when ill. Messages conveyed either risk information about (non-)compliance (the risk of non-compliance and the risk reduction of compliance), positive social outcomes of compliance (keeping others on campus healthy), or aimed to arouse empathy for vulnerable people on campus (protect people with fragile health). All posters had the same layout that stated the health hazard on top (‘the virus season has started’), followed by directions to comply with one of the two behavioral measures (either cough and sneeze into your elbow, or stay at home if you are ill), accompanied by a visualization. Then one of the three persuasive strategies followed (risk information, positive social outcomes, or arousing empathy), and finally the main goal was stated at the bottom of the poster (‘prevent the spread of viruses’).

Message phrasing and poster design were done in consultation with policy and communication staff from the Netherlands Ministry of Public Health, Welfare and Sports, to ensure close resemblance to messages used in practice by the government during the COVID-19 pandemic, and to maximize potential practical usability in the future. Students from one of the participating educational institutions also provided input via informal review to ensure message understandability and attractiveness. The three persuasive information strategies in the messages were selected based on the results of an online experiment conducted prior to the field study in which seven information types were tested (manuscript in preparation; study publicly pre-registered at the Open Science Framework (Van der Heijden et al., 2023a)).

### Measures

Variables were measured at both the pre- and post-exposure questionnaires, unless noted otherwise. A complete overview of all measures, including example items and substantiation, is included in Supplementary File 2. Recall of exposure frequency (measured only post-exposure; pragmatically categorized as 0, 1–2, 3–4, or 5+ times exposed) was the main independent variable. Outcome measures were assessed using five-point Likert scales or semantic differential scales (unless otherwise indicated) and encompassed intention to comply with the allocated behavioral measure; attitude, social norms, moral norm, self-efficacy, and response efficacy regarding the behavioral measure; actual compliance with this measure (answer options dichotomized into ‘*compliant*’ or ‘*not compliant*’); and perceived risk of COVID-19 and the flu. Intention to comply with the behavioral measure in a hypothetical high-risk pandemic situation was also assessed at the post-exposure questionnaire.

Assessed potential effect modifiers included trust in government, prosocial orientation (self- or other-oriented), perceived health status, knowledge of respiratory viruses and preventive measures (six statements that could be answered with ‘*true*’, ‘*not true*’ and ‘*don’t know*’), demographic characteristics (gender, age, ethnic background), educational institution, role (student/employee), behavioral measure type the participant was allocated to in the questionnaire, and whether the participant completed both T0 and T1 questionnaires or only T1.

### Data analysis

Outcome measures and effect modifiers were included as continuous variables (scale scores comprised the mean of scale items), except behavioral compliance which was categorical (compliant or not). For knowledge, the sum of correct answers was used. Effect modifiers were dichotomized before inclusion in the analyzes or otherwise categorized (all categories displayed in results tables). Reliability of all multi-item scales was established for all measures assessed with Likert or semantic differential response scales. All showed sufficient internal consistency, Cronbach's *α* ranging from .721 to .937.

Two-way multivariate analyzes of variance (MANOVA) were conducted to test the main effect of exposure frequency to persuasive messages on intention to comply (main outcome) and other behavioral determinants at T1, including the potential interaction effect with behavioral measure type. Interaction effects with other potential effect modifiers were examined through eleven additional MANOVAs. Logistic regression analysis assessed whether behavioral compliance at T1 was related to exposure frequency. Repeated measures ANOVA compared participants' intention to comply in the present low-risk pandemic situation (T1) to their anticipated intention to comply in a hypothetical high-risk situation. For participants who completed both T0 and T1, repeated measures MANOVA explored the extent to which changes in intention and behavioral determinants between T0 and T1 differed as a function of exposure frequency. Behavioral measure type was included as covariate in all analyzes. All analyzes were conducted using data of participants without any missing values on the relevant variables. Due to forced responses in the questionnaires (i.e. participants could only proceed after providing an answer to the previous question), there were very few missing variables per questionnaire.

Effects were considered statistically significant at *p* < .05. If a multivariate main or interaction effect on the combined outcome measures was found, this effect was further explored with univariate analyzes of the individual outcome measures. Due to space limitations, concise results are presented. Full information on all models, including descriptives and test statistics, is included in Supplementary File 3.

## Results

### Participant characteristics

**Figure 3.**
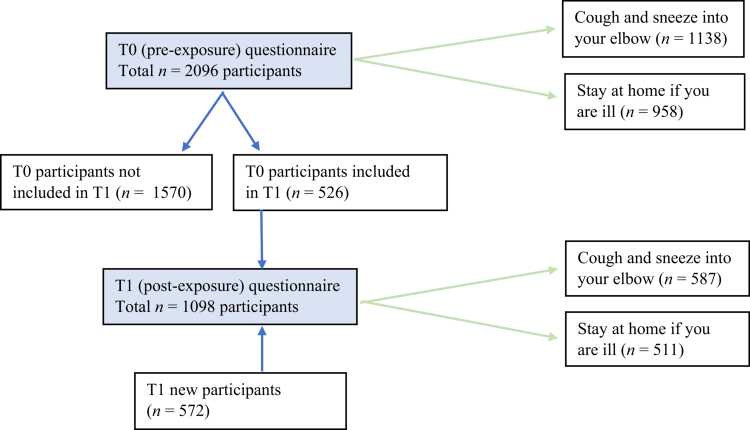
Participant flow and allocation to behavioral measures in the T0 and T1 questionnaires.

Characteristics of participants who completed the pre- (T0, *n* = 2096) and post-exposure (T1, *n* = 1098) questionnaires are shown in Table 1, stratified by educational institution. Of the T1 participants, *n* = 572 participated only at T1; *n* = 526 had also participated at T0. Of the participants who completed both questionnaires, *n =* 197 answered questions about the same behavioral measure at both times (randomly allocated). Figure 3 displays the participant flow and allocation to behavioral measures in the T0 and T1 questionnaires. The majority of participants were women (74.1% at T1), students (68.4% at T1), and 16–24 years of age (63.0% at T1). Just over half of participants at T1 (51.5%) had a Dutch background, 28.7% had a background from another Western country, and 19.5% had a background from a non-Western country. The composition of participants regarding age groups and ethnic background was significantly different in T1 compared to T0 (both *p*'s < .001), while the composition of participants regarding gender remained marginally stable (*p* = .051).

**Table 1.** Participant characteristics.

T0 (pre-exposure)	Role *n* (%)	Gender *n* (%)	Age (*M* years ± *SD,* range) Age group *n* (%)	Ethnic background^1^ *n* (%)
Total (*n* = 2096; 100%)	Student: 1922 (91.7%) Employee: 174 (8.3%)	Men: 552 (26.3%) Women: 1494 (71.3%) Other: 28 (1.3%) Prefer not to say: 22 (1.0%)	*M* = 22.2 years ***±*** 6.9; 16–65 16–24 years: 1772 (84.5%) 25–39 years: 234 (11.2%) 40–59 years: 80 (3.8%) 60+ years: 10 (0.5%)	Netherlands: 829 (39.6%) Non-western: 592 (28.2%) Western: 661 (31.5%) Missing: 14 (0.7%)
University A(*n* = 1329; 63.4%)	Student: 1267 (95.3%) Employee: 62 (4.7%)	Men: 314 (23.6%) Women: 978 (73.6%) Other: 22 (1.7%) Prefer not to say: 15 (1.1%)	*M* = 21.1 years ***±*** 4.7; 17–64 16–24 years: 1191 (89.6%) 25–39 years: 116 (8.7%) 40–59 years: 21 (1.6%) 60+ years: 1 (0.1%)	Netherlands: 356 (26.8%) Non-western: 423 (31.8%) Western: 539 (40.6%) Missing: 11 (0.8%)
				
University B (*n* = 390; 18.6%)	Student: 353 (90.5%) Employee: 37 (9.5%)	Men: 116 (29.7%) Women: 267 (68.5%) Other: 5 (1.3%) Prefer not to say: 2 (0.5%)	*M* = 23.2 years ***±*** 6.9; 16–64 16–24 years: 309 (79.2%) 25–39 years: 66 (16.9%) 40–59 years: 12 (3.1%) 60+ years: 3 (0.8%)	Netherlands: 206 (52.8%) Non-western: 82 (21.0%) Western: 101 (25.9%) Missing: 1 (0.3%)
				
University of applied sciences (*n* = 253; 12.1%)	Student: 192 (75.9%) Employee: 61 (24.1%)	Men: 74 (29.2%) Women: 174 (68.8%) Other: 0 (0.0%) Prefer not to say: 5 (2.0%)	*M* = 27.3 years ***±*** 11.9; 16–65 16–24 years: 164 (64.8%) 25–39 years: 45 (17.8%) 40–59 years: 39 (15.4%) 60+ years: 5 (2.0%)	Netherlands: 179 (70.8%) Non-western: 55 (21.7%) Western: 17 (6.7%) Missing: 2 (0.8%)
				
Intermediate vocational education (*n* = 124; 5.9%)	Student: 110 (88.7%) Employee: 14 (11.3%)	Men: 48 (38.7%) Women: 75 (60.5%) Other: 1 (0.8%) Prefer not to say: 0 (0.0%)	*M* = 21.2 years ***±*** 8.7; 16–63 16–24 years: 108 (87.1%) 25–39 years: 7 (5.6%) 40–59 years: 8 (6.5%) 60+ years: 1 (0.8%)	Netherlands: 88 (71.0%) Non-western: 32 (25.8%) Western: 4 (3.2%)
T1 (post-exposure)	Role *n* (%)	Gender *n* (%)	Age (*M* years *± SD,* range) Age group *n* (%)	Ethnic background^1^ *n* (%)
Total (*n* = 1098; 100%)	Student: 751 (68.4%) Employee: 347 (31.6%)	Men: 257 (23.4%) Women: 814 (74.1%) Other: 15 (1.4%) Prefer not to say: 12 (1.1%)	*M* = 27.2 years ***±*** 12.1; 16–67 16–24 years: 692 (63.0%) 25–39 years: 226 (20.6%) 40–59 years: 153 (13.9%) 60+ years: 27 (2.5%)	Netherlands: 565 (51.5%) Non-western: 214 (19.5%) Western: 315 (28.7%) Missing: 4 (0.4%)
University A (*n* = 544; 49.5%)	Student: 454 (83.5%) Employee: 90 (16.5%)	Men: 128 (23.5%) Women: 404 (74.3%) Other: 6 (1.1%) Prefer not to say: 6 (1.1%)	*M* = 23.0 years ***±*** 7.7; 17–64 16–24 years: 424 (77.9%) 25–39 years: 91 (16.7%) 40–59 years: 27 (5.0%) 60+ years: 2 (0.4%)	Netherlands: 173 (31.8%) Non-western: 133 (24.4%) Western: 238 (43.8%)
				
University B (*n* = 280; 25.5%)	Student: 170 (60.7%) Employee: 110 (39.3%)	Men: 62 (22.1%) Women: 208 (74.3%) Other: 7 (2.5%) Prefer not to say: 3 (1.1%)	*M* = 28.8 years ***±*** 12.6; 17–67 16–24 years: 155 (55.4%) 25–39 years: 75 (26.8%) 40–59 years: 40 (14.3%) 60+ years: 10 (3.6%)	Netherlands: 184 (65.7%) Non-western: 36 (12.9%) Western: 58 (20.7%) Missing: 2 (0.7%)
University of applied sciences (*n* = 211; 19.2%)	Student: 72 (34.1%) Employee: 139 (65.9%)	Men: 50 (23.7%) Women: 158 (74.9%) Other: 1 (0.5%) Prefer not to say: 2 (0.9%)	*M* = 37.3 years ***±*** 14.3; 16–66 16–24 years: 60 (28.4%) 25–39 years: 56 (26.5%) 40–59 years: 81 (38.4%) 60+ years: 14 (6.6%)	Netherlands: 160 (75.8%) Non-western: 33 (15.6%) Western: 16 (7.6%) Missing: 2 (0.9%)
				
Intermediate vocational education (*n* = 63; 5.7%)	Student: 55 (87.3%) Employee: 8 (12.7%)	Men: 17 (27.0%) Women: 44 (69.8%) Other: 1 (1.6%) Prefer not to say: 1 (1.6%)	*M* = 22.0 years ***±*** 10.9; 16–63 16–24 years: 53 (84.1%) 25–39 years: 4 (6.3%) 40–59 years: 5 (7.9%) 60+ years: 1 (1.6%)	Netherlands: 48 (76.2%) Non-western: 12 (19.0%) Western: 3 (4.8%)

Note: ^1^Netherlands indicates no migration background; Western indicates a Western migration background, i.e. origin from a European country (excluding Turkey), North-America, Oceania, Indonesia and Japan; Non-western indicates a non-western migration background, i.e. origin from a country in Africa, Latin-America, Asia (excluding Indonesia and Japan), or Turkey, following the classification from Statistics Netherlands (CBS, 2022).

### Associations between exposure frequency, intention to comply, and behavioral determinants

MANOVA was conducted to test hypothesis 1. Exposure frequency (IV) had a significant multivariate main effect on the combined outcome measures, *F*(24, 3150) = 2.01, *p* = .002; Wilks' *Λ* = 0.957. Univariate effects showed significant effects of exposure frequency on social norm (*F*(3, 1093) = 2.77, *p* = .041, $\eta_{\text{p}}^{2}$ = .008) and perceived risk of COVID-19 (*F*(3, 1093) = 5.04, *p* = .002, $\eta_{\text{p}}^{2}$ = .014) and the flu (*F*(3, 1093) = 3.97, *p* = .008, $\eta_{\text{p}}^{2}$ = .011), but not on intention to comply with the behavioral measures (*F*(3, 1093) = 1.98, *p* = .116, $\eta_{\text{p}}^{2}$ = .005) (Table 2). Hypothesis 1, that participants who are more frequently exposed to persuasive messages would have a relatively higher intention to comply, is thus not supported by the findings.

**Table 2.** Univariate effects of exposure frequency on intention to comply and behavioral determinants.

	Exposure frequency										
	0 times (*n* = 555)		1–2 times (*n* = 219)		3–4 times (*n* = 199)		5 + times (*n* = 125)				
	M	(SD)	M	(SD)	M	(SD)	M	(SD)	*F* (3, 1093)	*p*	η_p_^2^
Intention	3.51	1.23	3.79	1.20	3.66	1.29	3.66	1.21	1.98	.116	.005
Attitude	4.14	0.81	4.20	0.84	4.13	0.80	4.04	0.90	2.03	.108	.006
Social norm	**3.10** ^**a,b**^	0.92	**3.26** ^**a**^	0.83	**3.29** ^**b**^	0.83	3.20	0.89	2.77	.041	.008
Moral norm	3.30	1.10	3.51	1.04	3.48	0.98	3.36	1.03	2.38	.068	.006
Self-efficacy	3.51	1.18	3.77	1.06	3.60	1.17	3.59	1.11	2.19	.088	.006
Response efficacy	4.36	0.79	4.41	0.74	4.42	0.71	4.37	0.72	0.62	.604	.002
Perceived risk COVID-19	**2.93** ^**a,b**^	0.61	**3.06** ^**a**^	0.55	**3.10** ^**b**^	0.62	3.05	0.62	5.04	.002	.014
Perceived risk flu	**2.86** ^**a,b**^	0.58	**2.99** ^**a**^	0.53	**2.99** ^**b**^	0.57	2.92	0.53	3.97	.008	.011

Notes: (a) Significant differences within behavioral determinants as a function of exposure frequency are indicated with superscript and in bold within each respective row. (b) This table shows mean behavioral determinant scores of the two combined measures, i.e. coughing/sneezing into the elbow and staying home when ill. (c) *p < .05, **p < .01.

Behavioral measure type was included in the analysis as a potential effect modifier. The analysis revealed a significant multivariate main effect of behavioral measure type on the combined outcome variables: scores for coughing/sneezing into the elbow were consistently higher than for staying home when ill, *F*(8, 1083) = 111.61, *p* < .001; Wilks' *Λ* = 0.548. Univariate analyzes showed significant effects of behavioral measure type on all individual outcomes (all *p'*s < .05), except perceived risk of COVID-19 (*p* = .196) (Table 3). No significant interaction between exposure frequency and behavioral measure type on the combined outcome variables was identified, *F*(24, 3141) = 1.31, *p* = .142; Wilks' *Λ* = 0.972. However, as also shown in Table 3, exposure frequency only significantly impacted individual outcomes within the behavioral measure staying home when ill, and not within coughing/sneezing into the elbow.

**Table 3.** Means and standard deviations of scores on individual outcome measures stratified by behavioral measure type, and univariate main and interaction effects on individual outcome measures.

	Exposure frequency												
	0 times (*n* = 555)		1–2 times (*n* = 219)		3–4 times (*n* = 199)		5 + times (*n* = 125)		Total (*n* = 1098)				
	M	(SD)	M	(SD)	M	(SD)	M	(SD)	M	(SD)			
*Coughing and sneezing into elbow (n = 587)*													
Intention	4.17	1.01	4.23	1.05	4.40	0.89	4.18	1.04	4.23^**1**^	1.00			
Attitude	4.55	0.67	4.50	0.68	4.53	0.63	4.42	0.76	4.52^**2**^	0.68			
Social norm	3.25	0.91	3.45	0.78	3.51	0.73	3.42	0.77	3.36^**3**^	0.84			
Moral norm	3.48	1.02	3.69	1.00	3.70	0.92	3.53	0.99	3.57^**4**^	1.00			
Self-efficacy	4.33	0.67	4.31	0.71	4.34	0.77	4.21	0.69	4.31^**5**^	0.70			
Response efficacy	4.20	0.80	4.28	0.74	4.28	0.76	4.29	0.73	4.24^**6**^	0.77			
Perceived risk COVID-19	2.93	0.64	3.07	0.53	3.17	0.61	3.07	0.61	3.02	0.61			
Perceived risk flu	2.85	0.57	3.03	0.53	3.06	0.57	2.97	0.53	2.94^**7**^	0.56			
*Staying home when ill with symptoms (n = 511)*													
Intention	**2.83** ^**a**^	1.05	**3.21** ^**a,b**^	1.15	**2.79** ^**b**^	1.14	2.92	1.06	2.90^**1**^	1.09			
Attitude	3.72	0.72	3.81	0.86	3.66	0.71	3.50	0.79	3.70^**2**^	0.76			
Social norm	2.94	0.90	3.01	0.82	3.02	0.87	2.90	0.96	2.97^**3**^	0.89			
Moral norm	3.11	1.14	3.27	1.04	3.21	0.99	3.12	1.05	3.16^**4**^	1.09			
Self-efficacy	**2.67** ^**a**^	0.97	**3.04** ^**a,b,c**^	1.02	**2.71** ^**b**^	0.94	**2.71** ^**c**^	0.99	2.75^**5**^	0.98			
Response efficacy	4.53	0.75	4.57	0.70	4.59	0.61	4.48	0.70	4.55^**6**^	0.71			
Perceived risk COVID-19	2.94	0.58	3.04	0.58	3.02	0.64	3.03	0.63	2.98	0.60			
Perceived risk flu	2.87	0.58	2.94	0.54	2.90	0.56	2.86	0.54	2.89^**7**^	0.57			
*Behavioral measure type allocated in the questionnaire (coughing and sneezing or staying home)*											*F* (1, 1090)	*p*	$\eta_{\text{p}}^{2}$
Intention											319.70	<.001**	0.227
Attitude											272.65	<.001**	0.200
Social norm											52.30	<.001**	0.046
Moral norm											33.21	<.001**	0.030
Self-efficacy											656.26	<.001**	0.376
Response efficacy											28.56	<.001**	0.026
Perceived risk COVID-19											1.67	.196	0.002
Perceived risk flu											4.86	.028*	0.004
Exposure frequency * Behavior type allocated in the questionnaire											*F* (3, 1090)	*p*	$\eta_{\text{p}}^{2}$
Intention											2.84	.037*	.008
Attitude											0.96	.412	.003
Social norm											0.94	.422	.003
Moral norm											0.16	.926	.000
Self-efficacy											2.94	.032*	.008
Response efficacy											0.33	.805	.001
Perceived risk COVID-19											0.95	.418	.003
Perceived risk flu											1.45	.228	.004

Notes: (a) Significant differences in behavioral determinant scores between coughing/sneezing into the elbow and staying home when ill are indicated with a number in superscript (1–7) within the ‘Total’ column of each behavioral measure. (b) Significant differences within behavioral determinants as a function of the interaction between exposure frequency and behavioral measure type are indicated in bold superscript within each respective row. (c) *p < .05, **p < .01.

Furthermore, regarding univariate effects on individual outcomes (also shown in Table 3), it should be noted that although both the main effects of behavioral measure type and some of the interaction effects with exposure frequency on individual outcomes are statistically significant, their strengths differ. The large effect size for behavioral measure type (e.g. $\eta_{\text{p}}^{2}$ = .227 for intention) suggests a substantial effect, whereas the small effect size for the interaction between exposure frequency and behavioral measure type (e.g. $\eta_{\text{p}}^{2}$ = .008 for intention) suggests a weak effect.

### Main and interaction effects of potential effect modifiers

To explore research question 1, MANOVAs were performed with exposure frequency and each respective potential effect modifier as IVs in the analyzes, including behavioral measure type as a covariate. All potential effect modifiers showed multivariate main effects on the combined outcomes (all *p*'s < .05) (Table 4). Moreover, univariate analyzes of each potential effect modifier on the individual outcomes (Table 5) showed a significant effect of gender, i.e. women had higher scores than men on almost all behavioral determinants: intention (*F*(1, 1062) = 11.21, *p* < .001, $\eta_{\text{p}}^{2}$ = .010), attitude (*F*(1, 1062) = 7.41, *p* = .007, $\eta_{\text{p}}^{2}$ = .007), moral norm (*F*(1, 1062) = 5.77, *p* = .016, $\eta_{\text{p}}^{2}$ = .005), perceived risk of COVID-19 (*F*(1, 1062) = 24.88, *p* < .001, $\eta_{\text{p}}^{2}$ = .023), and perceived risk of the flu (*F*(1, 1062) = 30.79, *p* < .001, $\eta_{\text{p}}^{2}$ = .028). Furthermore, ethnic background significantly impacted social and moral norm, as participants with a Dutch background had lower social norm (*F*(2, 1081) = 6.20, *p* = .002, $\eta_{\text{p}}^{2}$ = .011) and moral norm scores (*F*(2, 1081) = 10.57, *p* < .001, $\eta_{\text{p}}^{2}$ = .019) than participants with migration backgrounds. Moreover, participants with a non-western migration background perceived a higher risk of COVID-19 (*F*(2, 1081) = 21.30, *p* < .001, $\eta_{\text{p}}^{2}$ = .038) and the flu (*F*(2, 1081) = 25.22, *p* < .001, $\eta_{\text{p}}^{2}$ = .045) than people of other backgrounds.

**Table 4.** Multivariate main effects of demographic and personal factors on the combined outcome measures.

Moderator	*F* (df)	*p*	Wilks' Λ
Educational institution (university; uni. of appl. sciences; interm. voc. edu.)	*F* (16, 2156) = 2.88	<.001**	0.959
Role (student or employee)	*F* (8, 1082) = 5.167	<.001**	0.963
Participation in T0 (yes or no)	*F* (8, 1082) = 2.638	=.007**	0.981
Gender (man or woman)	*F* (8, 1055) = 5.938	<.001**	0.957
Age (16–24; 25–39; 40–59; 60 + years)	*F* (24, 3116) = 2.721	<.001**	0.942
Ethnic background (Netherlands; Non-western migration background; Western migration background)	*F* (16, 2148) = 6.267	<.001**	0.913
Knowledge of respiratory viruses and preventive measures (low or high)	*F* (8, 1082) = 8.760	<.001**	0.939
Trust in government (low or high)	*F* (8, 1082) = 2.396	=.015*	0.983
Prosocial orientation (self or others)	*F* (8, 1082) = 4.619	<.001**	0.967
Perceived health status (bad or good)	*F* (8, 1082) = 8.086	<.001**	0.944

Notes: (a) A separate model was run for each potential moderator. (b) Exposure frequency as main independent variable and behavioral measure type as covariate were included in all models. (c) No multivariate interaction effects between exposure frequency and demographic and personal factors on the combined outcome measures were identified (all *p*'s > .05). (d) *p < .05, **p < .01.

**Table 5.** Univariate main and interaction effects of personal and demographic factors ( × exposure frequency) on individual outcome measures.

Moderator	Outcome measure^b^		*F* (df)		*p*	$\eta_{\text{p}}^{2}$	Effect specification
Educational institution (university; uni. of appl. sciences; interm. voc. edu.)		Social norm Moral norm Response efficacy		*F* (2, 1085) 5.86 5.13 3.04	.003** .006** .048*	0.011 0.009 0.006	Interm. voc. edu. (*M =* 3.66*, SD* = 0.88) > uni. appl. sci. (*M* = 3.11, *SD* = 0.82) > university (*M* = 3.16, *SD* = 0.89) University (*M* = 3.41, *SD* = 1.06) > uni. appl. sci. (*M* = 3.28, *SD* = 1.07) > interm. voc. edu. (*M* = 3.27, *SD* = 0.98) None ^d^
Gender (man or woman)		Intention Attitude Moral norm Perc. risk COVID-19 Perceived risk flu		*F* (1, 1062) 11.21 7.41 5.77 24.88 30.79	<.001** .007* .016* <.001** <.001**	0.010 0.007 0.005 0.023 0.028	Women (*M* = 3.66, *SD* = 1.20) > men (*M* = 3.44, *SD* = 1.35) Women (*M* = 4.17, *SD* = 0.80) > men (*M* = 4.03, *SD* = 0.87) Women (*M* = 3.43, *SD* = 1.04) > men (*M* = 3.18, *SD* = 1.11) Women (*M* = 3.06, *SD* = 0.59) > men (*M* = 2.80, *SD* = 0.62) Women (*M* = 2.98, *SD* = 0.54) > men (*M* = 2.71, *SD* = 0.59)
Gender × exposure frequency (man or woman; 0, 1–2, 3–4, 5+ times)		Intention		*F* (3, 1062) 4.34	.005**	0.012	Women: Exposed 5 + times (*M* = 3.86, *SD* = 1.12) > 0 times (*M* = 3.53, *SD* = 1.19) Men: Exposed 1-2 times (*M* = 3.69, *SD* = 1.20) > 0 times (*M* = 3.44, *SD* = 1.37) > 3–4 times (*M* = 3.41 *SD* = 1.48) > 5 + times (*M* = 3.03, *SD* = 1.26)
Age (16–24; 25–39; 40–59; 60+ years)		Social norm		*F* (3, 1081) 3.23	.022*	0.009	Aged 16–24 years (*M* = 3.23, *SD* = 0.92) > 25–39 years (*M* = 3.03, *SD* = 0.82)
Ethnic background (Netherlands; Non-western migration background; Western migration background)		Social norm Moral norm Perc. risk COVID-19 Perceived risk flu		*F* (2, 1081) 6.20 10.57 21.30 25.22	.002** <.001** <.001** <.001**	0.011 0.019 0.038 0.045	Netherlands (*M* = 3.09, *SD* = 0.89) < Western (*M* = 3.27, *SD* = 0.86) < Non-western (*M* = 3.27, *SD* = 0.89) Netherlands (*M* = 3.25, *SD* = 1.04) < Western (*M* = 3.56, *SD* = 1.07) < Non-western (*M* = 3.43, *SD* = 1.06) Non-Western (*M* = 3.20, *SD* = 0.64) > Netherlands (*M* = 2.92, *SD* = 0.56) > Western (*M* = 3.01, *SD* = 0.62) Non-Western (*M* = 3.12, *SD* = 0.58) > Netherlands (*M* = 2.84, *SD* = 0.51) > Western (*M* = 2.93, *SD* = 0.61) Western (*M* = 2.93, *SD* = 0.61) > Netherlands (*M* = 2.84, *SD* = 0.51)
Ethnic background * exposure frequency (Netherlands; Non-western migration background; Western migration background; 0, 1–2, 3–4, 5+ times)		Perc. risk COVID-19 Perceived risk flu		*F* (6, 1081) 3.10 2.42	.005** .025*	0.017 0.013	Netherlands: Exposed 0 times (*M* = 2.84, *SD* = 0.58) < 1–2 times (*M* = 3.02, *SD* = 0.50) < 3–4 times (*M* = 3.00, *SD* = 0.57) Non-western: Exposed 0 times (*M* = 3.04, *SD* = 0.61) < 3–4 times (*M* = 3.53, *SD* = 0.60) < 5 + times (*M* = 3.45, *SD* = 0.70 Exposed 1–2 times (*M* = 3.14, *SD* = 0.56) < 3-4 times (*M* = 3.53, *SD* = 0.60) < 5 + times (*M* = 3.45, *SD* = 0.70 Netherlands: Exposed 0 times (*M* = 2.78, *SD* = 0.54) < 1–2 times (*M* = 2.92, *SD* = 0.45) < 3–4 times (*M* = 2.91, *SD* = 0.51) Non-western: Exposed 0 times (*M* = 2.97, *SD* = 0.54) < 3–4 times (*M* = 3.34, *SD* = 0.64) < 5 + times (*M* = 3.33, *SD* = 0.54)
Role (student or employee)		Intention Social norm Self-efficacy		*F* (1, 1089) 8.71 4.32 5.09	.003** .038* .024*	0.008 0.004 0.005	Employee (*M* = 3.75, *SD* = 1.27) > student (*M* = 3.55, *SD* = 1.17) Student (*M* = 3.20, *SD* = 0.92) > employee (*M* = 3.13, *SD* = 0.79) Employee (*M* = 3.67, *SD* = 0.99) > student (*M* = 3.55, *SD* = 1.22)
Participation in T0 (yes or no)		Moral norm		*F* (1, 1089) 4.40	.036*	0.004	Yes (*M* = 3.40, *SD* = 1.07) > no (*M* = 3.36, *SD* = 1.06)
Knowledge of respiratory viruses and preventive measures (low or high)		Intention Attitude Moral norm Response efficacy		*F* (1, 1089) 7.72 18.11 6.78 42.47	.006** <.001** .009** <.001**	0.007 0.016 0.006 0.038	High (*M* = 3.63, *SD* = 1.23) > low (*M* = 3.55, *SD* = 1.26) High (*M* = 4.18, *SD* = 0.80) > low (*M* = 4.03, *SD* = 0.88) High (*M* = 3.44, *SD* = 1.05) > low (*M* = 3.21, *SD* = 1.07) High (*M* = 4.50, *SD* = 0.68) > low (*M* = 4.07, *SD* = 0.87)
Trust in government (low or high)		Response efficacy		*F* (1, 1089) 13.12	<.001**	0.012	High (*M* = 4.50, *SD* = 0.63) > low (*M* = 4.29, *SD* = 0.84)
Prosocial orientation (self or others)		Response efficacy Perc. risk COVID-19 Perceived risk flu		*F* (1, 1089) 7.25 15.00 15.88	.007** <.001** <.001**	0.007 0.014 0.014	Other-oriented (*M* = 4.44, *SD* = 0.72) > self-oriented (*M* = 4.30, *SD* = 0.81) Self-oriented (*M* = 3.10, *SD* = 0.66) > other-oriented (*M* = 2.94, *SD* = 0.56) Self-oriented (*M* = 3.00, *SD* = 0.61) > other-oriented (*M* = 2.87, *SD* = 0.53)
Perceived health status (bad or good)		Perc. risk COVID-19 Perceived risk flu		*F* (1, 1089) 41.53 54.71	<.001** <.001**	0.037 0.048	Bad (*M* = 3.24, *SD* = 0.66) > good (*M* = 2.92, *SD* = 0.56) Bad (*M* = 3.18, *SD* = 0.60) > good (*M* = 2.83, *SD* = 0.53)

Notes: (a) Exposure frequency as main independent variable and behavioral measure type as covariate were included in all models. Because there were no significant differences between both behavioral measure types, the combined scores of both behaviors are included. (b) Only significant effects are displayed in Table 5. Complete descriptives and test statistics of all models are provided in Supplementary File 1. (c) Although the univariate test indicated a significant effect, no significant difference was detected in the subsequent pairwise comparisons. (d) *p < .05, **p < .01.

No multivariate interaction effects were identified between exposure frequency and any of the potential effect modifiers on the combined outcomes (all *p*'s > .05). Univariate analyzes showed limited interaction effects between exposure frequency and potential effect modifiers on individual outcomes (Table 5). Intention to comply was significantly higher among women who were exposed five or more times than for women who were not exposed. For men, intention to comply was higher when exposed one or two times, compared to less or more times (*F*(3, 1062) = 4.34, *p* = .005, $\eta_{\text{p}}^{2}$ = .012). Among participants with Dutch and non-western migration backgrounds, perceived risk of COVID-19 and the flu was higher when exposed more frequently, compared to less or no exposure (COVID-19 *F*(6, 1081) = 3.10, *p* = .005, $\eta_{\text{p}}^{2}$ = .017, flu *F*(6, 1081) = 2.42, *p* = .025, $\eta_{\text{p}}^{2}$ = .013), although for participants with a Dutch background there was no significant difference between those who were exposed five times or more versus those who were not exposed.

### Behavioral compliance as a function of exposure frequency and behavioral measure type

To test hypothesis 2, logistic regression analysis was conducted with exposure frequency as independent variable, compliance with either of the behavioral measures as outcome, and behavioral measure type as covariate. The analysis showed no significant association between compliance with behavioral measures and exposure frequency (all *p*'s > .05) (Table 6). Hypothesis 2, that participants who are more frequently exposed to persuasive messages comply with behavioral measures more often than participants who are less frequent or not exposed, is not supported by the findings. However, the analysis showed a significant association between compliance and behavioral measure type. Participants were more likely to comply with coughing/sneezing into the elbow than with staying home when ill, *OR* = 0.165, 95% CI [0.117, 0.233]. Thus, the odds of compliance with staying home when ill were 83.5% lower compared to coughing and sneezing into the elbow.

**Table 6.** Logistic regression model coefficients and odds ratios of post-exposure behavioral compliance.

Independent variable^a^	*B (SE)*	*p*	OR (95% CI)
Exposed 0 times			1.00
Constant	0.36 (0.12)	.002	1.432
Exposed 1-2 times	0.12 (0.20)	.544	1.129 (0.764, 1.667)
Exposed 3-4 times	0.33 (0.20)	.103	1.393 (0.935, 2.076)
Exposed 5+ times	−0.06 (0.24)	.794	0.939 (0.587, 1.504)

Notes: (a) Compared to ‘no exposure’. (b) The model explains between 14.1% and 18.8% of the variance in compliance, *R*^*2*^ = 0.141 (Cox & Snell), *R*^*2*^ = 0.188 (Nagelkerke). (c) The model is a good fit for the data, *χ*^*2*^(4) = 126.30, *p* < .001 (Omnibus tests of model coefficients); *χ*^*2*^(5) = 0.69, *p* = .984 (Hosmer and Lemeshow). (d) *p <  .05, **p < .01.

### Changes over time in intention to comply and behavioral determinants

Repeated measures MANOVA was conducted with data from participants who completed the questionnaire at both T0 and T1, to explore research question 2. With exposure frequency as between-subjects factor, time (T0 or T1) as within-subjects factor, and behavioral measure type as covariate, the analysis showed a significant multivariate main effect of time on the combined outcomes, indicating changes in the outcomes from pre- to post-exposure (*F*(8, 185) = 3.15, *p* = .002; Wilks' *Λ* = 0.880). There was no significant interaction effect between time and exposure frequency on the combined outcomes, *F*(24, 537.158) = 1.17, *p* = .266; Wilks' *Λ* = 0.863. Univariate analyzes showed a significant main effect of time on intention to comply (decreasing from T0 to T1, *F*(1, 192) = 5.877, *p* = .016) and social norm (decreasing from T0 to T1, *F*(1, 192) = 17.332, *p* < .001). Thus, intention to comply and social norm were lower post-exposure than pre-exposure, regardless of exposure frequency. For attitude, a significant interaction effect between time and exposure frequency was found, *F*(3, 192) = 5.037, *p* = .002. Attitudes improved from T0 to T1 among unexposed participants, remained stable for those exposed 1–2 or 3–4 times, but declined for participants with five or more exposures. No pre-post changes were found for moral norm, self-efficacy, response efficacy, perceived risk of COVID-19, and perceived risk of the flu.

### Intention to comply in a hypothetical high-risk pandemic situation

Intention to comply in a hypothetical high-risk pandemic situation was compared to the present low-risk situation (both measured at T1) using repeated measures ANOVA, addressing research question 3. The analysis showed a main effect of pandemic situation (current low-risk or hypothetical high-risk) on intention to comply. Intention to comply was significantly higher in a hypothetical high-risk pandemic situation (*M* = 4.01, *SD* = 0.94) than in the current low-risk situation (*M* = 3.61, *SD* = 1.24, *F*(1, 1096) = 8.08, *p* = .005). Additionally, a significant interaction effect was found between pandemic situation and behavioral measure type (*F*(1, 1096) = 112.99, *p* < .001, $\eta_{\text{p}}^{2}$ = .093) (Table 7). Intention to comply was higher in the hypothetical high-risk than in the current low-risk situation for both behavioral measure types. However, the mean difference was substantially larger for staying home when ill (*MD* = 0.73, *p* < .001) than for coughing and sneezing into the elbow (*MD* = 0.11, *p* = .005). This reflects that intention to comply with coughing and sneezing into the elbow is already high in the current low-risk situation, and the potential for increase is smaller than for staying home when ill.

**Table 7.** Intention to comply in the current low-risk situation vs. in a hypothetical high-risk situation, stratified by behavioral measure type; and test statistics of the interaction between pandemic risk situation and behavioral measure type.

	Low-risk situation intention (current situation, T1)		Hypothetical high-risk situation intention				
	M	SD	M	SD	M difference		*p*
*Coughing and sneezing into elbow (n = 587)*	**4.23**	1.00	**4.34**	0.78	0.11		.005
*Staying home when ill with symptoms (n = 511)*	**2.90**	1.09	**3.63**	0.97	0.73		<.001
*Total (n = 1098)*	**3.61**	1.24	**4.01**	0.94			
					*F (1, 1096)*	*p*	$\eta_{\text{p}}^{2}$
Pandemic situation * Behavioral measure type					112.99	<.001**	0.093

Notes: (a) Significant differences in intention within each behavioral measure type are indicated in bold within each row. (b) *p < .05, **p < .01.

## Discussion

This field study tests the effects of persuasive messages on (behavioral determinants of) compliance with two behavioral measures to prevent the spread of viruses that cause respiratory infections such as COVID-19 and the flu, notably: coughing and sneezing into the elbow, and staying home when ill with respiratory infection symptoms. Exposure frequency has a significant effect on social norm regarding the behavioral measures and perceived risk of COVID-19 and the flu, but not on compliance or intention to comply. Hypothesis 1 and 2, i.e. that participants who are more frequently exposed to persuasive messages have a higher intention to comply and comply more often with behavioral measures than participants who are less frequent or not exposed, are thus not supported by the findings. As participants were exposed to messages conveying risks of non-compliance, positive social outcomes of compliance, and promoting empathy for others, it is to be expected that significant effects were found on related determinants.

### Effect modifiers of the association between exposure frequency and behavioral determinants

Insights are also gained regarding the three exploratory research questions. Regarding research question 1, exposure frequency significantly impacts individual outcomes within the behavioral measure staying home when ill, but not within coughing/sneezing into the elbow. Moreover, compliance with and behavioral determinants of staying home when ill are significantly lower than compliance with and determinants of coughing/sneezing into the elbow, regardless of exposure frequency. This suggests that the effectiveness of repeated exposure seems to depend on the type of behavioral measure targeted in the messages. More specifically, it suggests that people are more willing to comply with behavioral measures that incur less personal and social cost. Coughing and sneezing into the elbow hardly disrupts everyday life, whereas staying home when ill with respiratory infection symptoms may severely impede daily activities and may arouse resistance, especially when one is experiencing only mild symptoms. This interpretation is consistent with research reporting that behavioral measures with moderate personal or social cost (e.g. the number of visitors allowed in home settings during the COVID-19 pandemic is slightly restricted) were more effective in reducing the number of infections than behavioral measures with high personal or social cost (e.g. the number of allowed visitors is severely restricted), due to more non-compliance with high-cost measures (Wallinga et al., 2024).

### Changes over time (pre- to post-exposure) in intention to comply and behavioral determinants as a function of exposure frequency

Regarding research question 2, the findings can be cautiously interpreted by drawing on theory regarding the possible effects of repeated exposure to persuasive messages. This theorizing underscores that while exposure is necessary for persuasive messages to induce changes in behavior and determinants (Hornik, 2002), too much exposure can lead to undesired effects, such as message fatigue and reactance, counteracting changes in behavioral determinants and behavior (Cho & Salmon, 2007; Okuhara et al., 2023; Shen et al., 2022; Skurka & Keating, 2024; So et al., 2016; Zhao et al., 2023). The identified difference in intention scores between men and women depending on exposure frequency may be explained by higher reactance tendencies for men, who display the highest scores when exposed once or twice, but not more often. On the other hand, women show higher scores following more exposure. Whether reactance is a relevant factor for men, and whether even more exposure produces more positive effects for women needs to be examined in further studies. A similar pattern is found for perceived risk of COVID-19 and the flu, where participants with a non-Western migration background display the highest perceived risk with five or more exposures, whereas for Dutch participants the highest perceived risk score is obtained with one to four rather than more exposures. More research is needed to find factors that might explain this difference. No other interaction effects between exposure frequency and demographic characteristics, trust in government, prosocial orientation, perceived health, educational institution, student/employee, and participation in pre-exposure questionnaire are identified.

### Intention to comply in a hypothetical high-risk pandemic situation versus the current low-risk situation

Finally, regarding research question 3, it is observed that for both behavioral measures, intention to comply following exposure to persuasive messages is higher in a hypothetical high-risk pandemic situation than in the current low-risk situation. In both situations intention to comply with coughing/sneezing into the elbow is higher than intention of staying home when ill; however, the increase in intention to comply between the low- and high-risk situation is much larger for staying home when ill than for coughing/sneezing into the elbow. This reflects that intention to comply with coughing/sneezing into the elbow is already high in the low-risk situation, and thus the potential for increase is smaller than for staying home when ill. In addition, the felt need to act, including willingness to comply with a measure that entails more personal or social cost, likely increases when the risk of infection and experiencing severe symptoms is higher (cf. e.g. Kafadar et al., 2022; Limbu & Gautam, 2023).

### Strengths and limitations

The study has several strengths, yet is also prone to some limitations that warrant cautious interpretation and generalization of the findings. A strength of the field study design is the high external validity, as evidence is provided on the effects of persuasive messages on (determinants of) behavioral compliance in real-life practice. A related limitation is that field studies are susceptible to external influences beyond the researchers' control, e.g. seasonal illness trends or simultaneous public health campaigns, which may have resulted in confounding. Another study strength is the inclusion of a broad range of participants, representing all common post-secondary school educational levels in the Netherlands.

Recruitment and study conduct at the intermediate vocational education institution posed more challenges. Participants at the intermediate vocational education institution only received questions about coughing and sneezing into the elbow, as they were also only exposed to messages about this measure, per request of the educational institution. Thus, it remains opaque to what extent findings regarding staying home when ill are also applicable in these participants.

Moreover, the number of participants from this institution is relatively low. Consequently, the number of participants is not equally divided over all participating educational institutions, which poses another limitation: unequal participant numbers in groups potentially influence identification of statistically significant effects. Participant numbers were also unequal for message exposure frequency, with lower numbers of participants reporting higher exposure frequency, and for behavioral type, with lower numbers of participants in the group that answered questions about staying home when ill. We note that this may have affected statistical power and robustness of results.

Moreover, differences in the demographic composition of participants in T1 compared to T0 need to be taken into account when interpreting the results. Furthermore, self-report questionnaires entail a risk of socially desirable responding, and a risk of recall bias. Notably, the variable exposure frequency reflects participants' reported estimates of how frequently they were exposed to the persuasive messages, but they might not have been able to recall this correctly. There might be underreporting because they do not recall exposure and recall is more likely when the messages are mentally processed.

A final study strength as well as potential limitation comprises the message phrasing and poster design. Message phrasing and poster design were co-created with policy and communication experts of the Netherlands Ministry of Public Health, Welfare and Sports, as well as with students. This was done to ensure messages resembled communication of the Netherlands government during the COVID-19 pandemic, and to maximize potential practical usability in the future. However, tailoring the materials to the context of the Netherlands may also limit generalizability.

### Recommendations

Lack of effects on intention and behavior might be due to the low-risk pandemic situation during the present field study, in which risk awareness is low, and thus is the felt need to act. As it is also observed that in a hypothetical high-risk pandemic situation the intention to comply increases, it might be expected that exposure to persuasive messages a high-risk situation is potentially more effective in enhancing intention and behavior. In addition, it is recommended to tailor persuasive messages to the needs of specific subgroups (e.g. by gender or cultural background, as relevant differences were found in this study) and to combine effective communication strategies such as persuasive messages with structural or policy measures, to optimize potential effects on compliance. Finally, based on the finding that people are more willing to comply with measures that are relatively easy to perform and require less personal sacrifice, explanations of specific measures' relevance and effectiveness (rather than a one-size-fits-all-measures approach) and practical support for people to comply with more difficult to perform behaviors might further stimulate compliance. Future research could investigate this, as well as the effectiveness of persuasive messages when exposure periods are longer, messages are ubiquitously present (rather than only in one specific setting), and when there is a real high-risk pandemic situation. Moreover, future research could benefit from methodological refinement such as using behavioral outcomes and objective measures of exposure rather than self-report measures.

## Conclusion

In conclusion, exposure to persuasive messages impacts those behavioral determinants at which these messages are directed, that is, social norms and perceived risk of COVID-19 and the flu. However, this did not lead to changes in compliance nor intention to comply with behavioral measures. This indicates that exposing people to concise persuasive messages in their digital and physical environment, similar to communication deployed by, e.g. the Dutch government during the COVID-19 pandemic, initiates the process for enhancing compliance but as such is not yet sufficient. The findings imply practical implications and provide leads for future research.

## Supplementary Material

Supplementary_file 1_Persuasive_messages.pdf Supplementary_file 1_Persuasive_messages.pdf

Supplementary_file_3_MANOVAs_descriptives_test_statistics.pdf Supplementary_file_3_MANOVAs_descriptives_test_statistics.pdf

Supplementary_file_2_Overview_measures.pdf Supplementary_file_2_Overview_measures.pdf

## Data Availability

The data that support the findings of this study are available from the corresponding author upon reasonable request.
