# Supplementary material for: ‘Cough and sneeze into your elbow’: a field study testing the effects of persuasive messages on compliance with behavioral measures to prevent the spread of respiratory viruses
Source: Health Psychol Behav Med. 2026 Jan 20;14(1):2616931. doi: 10.1080/21642850.2026.2616931 (PMC12821337; doi:10.1080/21642850.2026.2616931)
Supplement: Supplementary_file 1_Persuasive_messages.pdf [file RHPB_A_2616931_SM8641.pdf]

THE VIRUS SEASON HAS STARTED

STAY AT HOME IF YOU ARE ILL

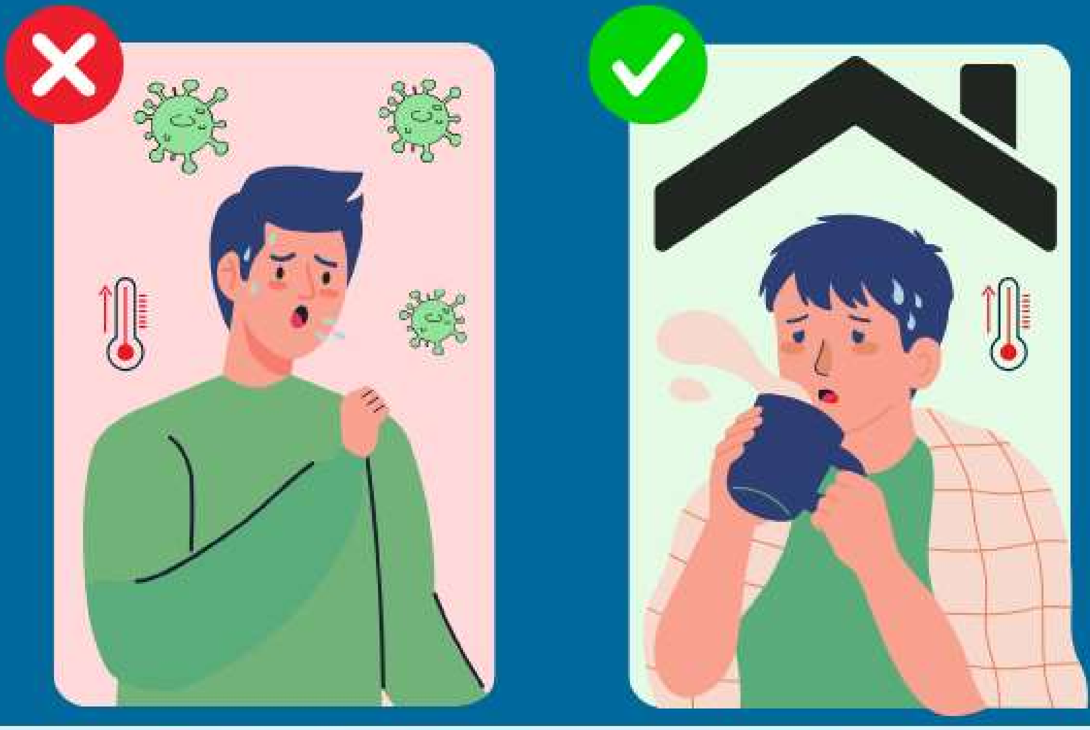

"My asthma means: being extra cautious of viruses."

Sam, 20

PROTECT PEOPLE LIKE SAM

Prevent the spread of viruses

THE VIRUS SEASON HAS STARTED

COUGH AND SNEEZE INTO YOUR ELBOW

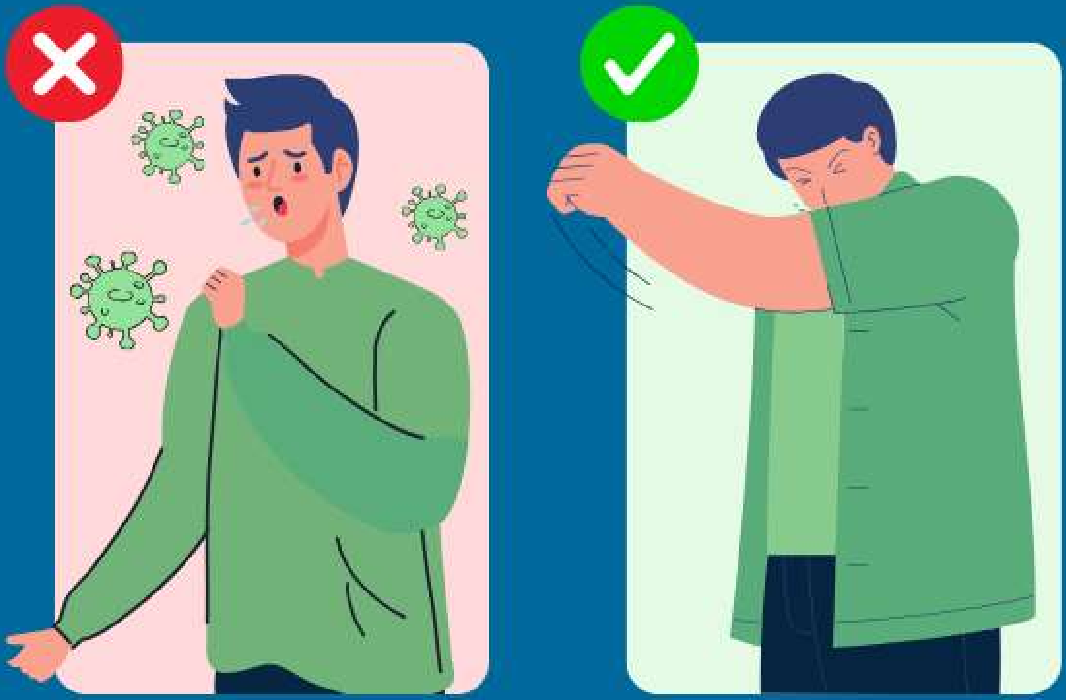

"My asthma means: being extra cautious of viruses."
Sam, 20

PROTECT PEOPLE LIKE SAM

Prevent the spread of viruses

THE VIRUS SEASON HAS STARTED

STAY AT HOME IF YOU ARE ILL

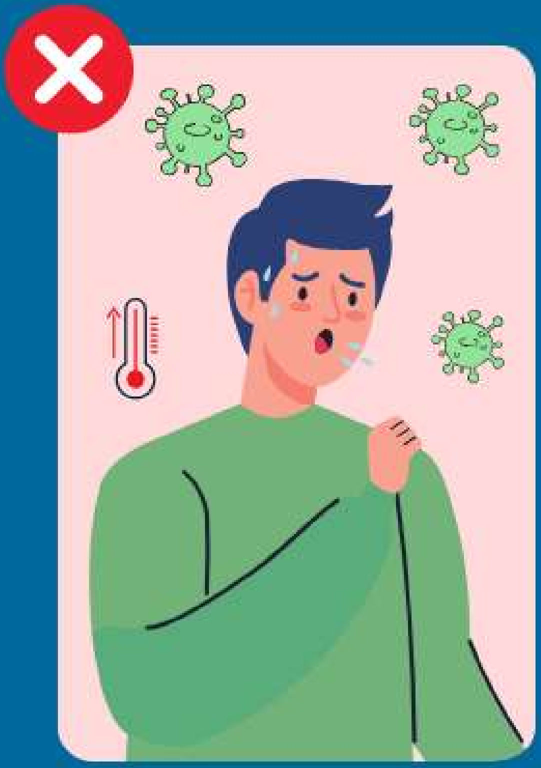

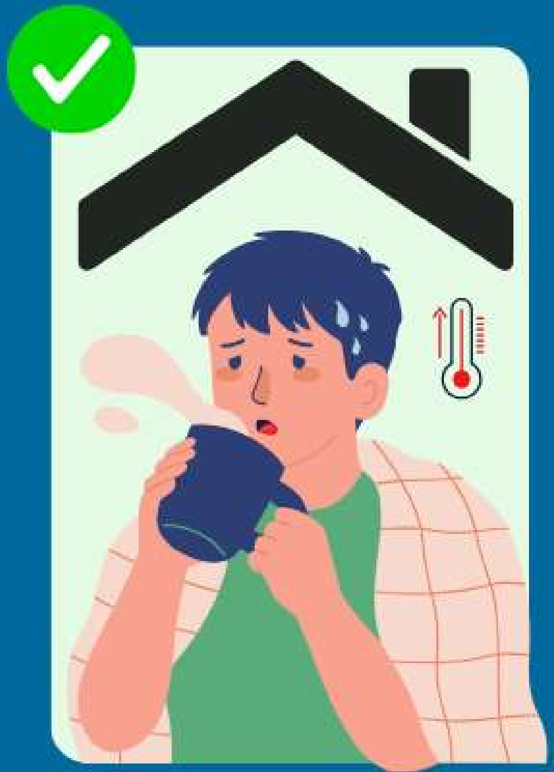

KEEP OTHERS ON CAMPUS HEALTHY

Prevent the spread of viruses

THE VIRUS SEASON HAS STARTED

COUGH AND SNEEZE INTO YOUR ELBOW

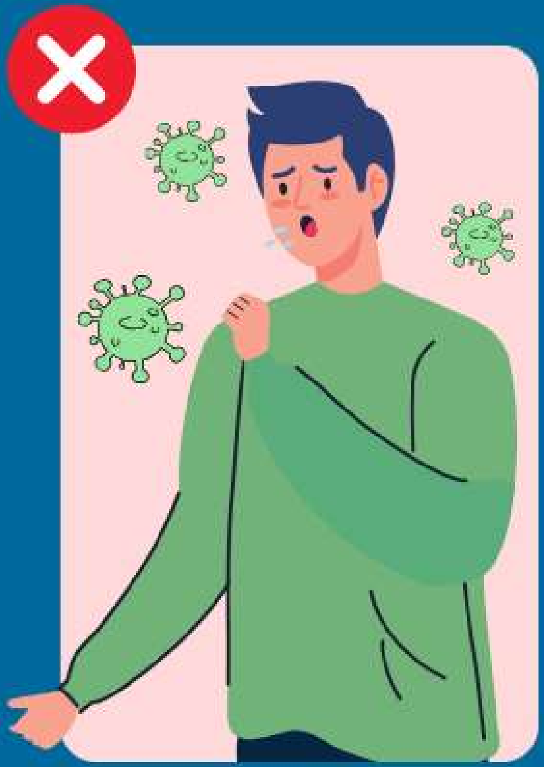

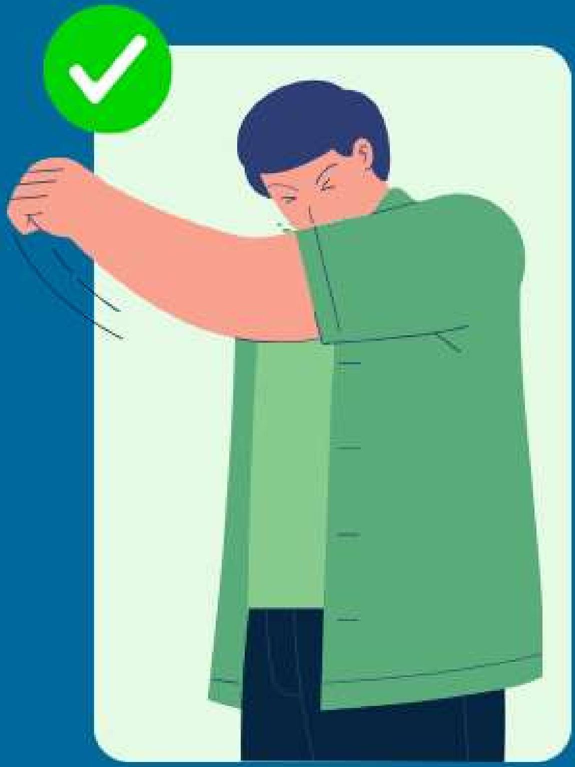

KEEP OTHERS ON CAMPUS HEALTHY

Prevent the spread of viruses

THE VIRUS SEASON HAS STARTED

STAY AT HOME IF YOU ARE ILL

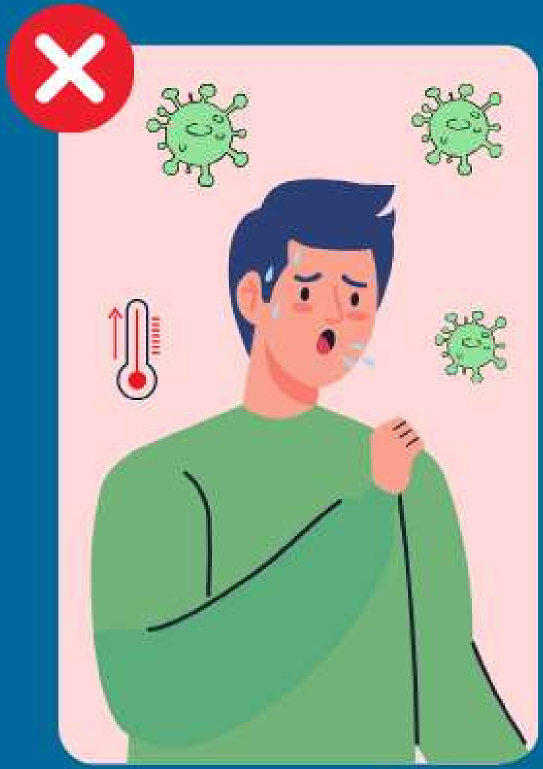

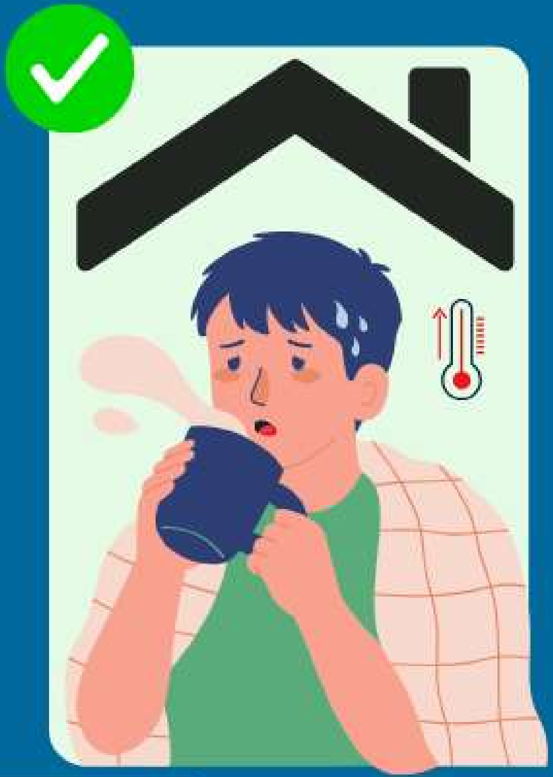

Higher risk

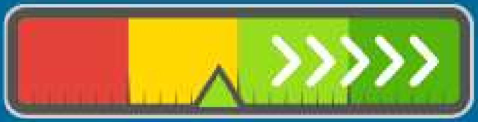

Lower risk

LOWER THE RISK OF INFECTING OTHERS

Prevent the spread of viruses

THE VIRUS SEASON HAS STARTED

COUGH AND SNEEZE INTO YOUR ELBOW

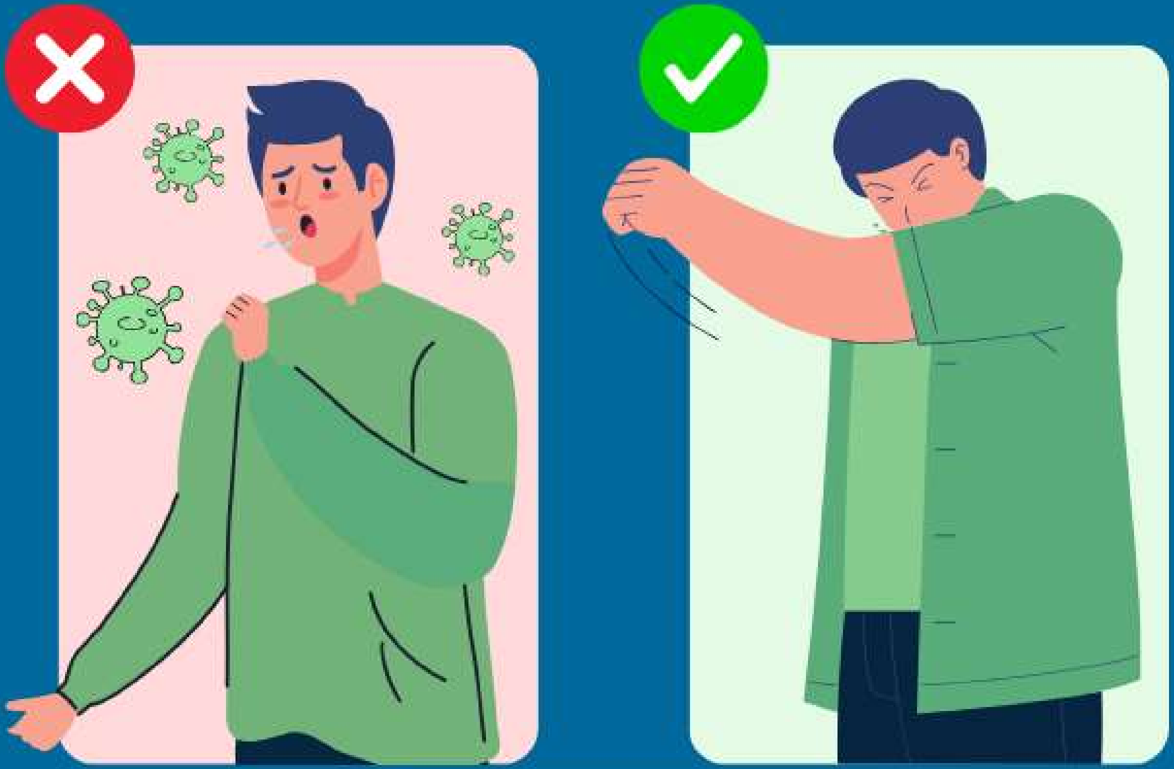

Higher risk 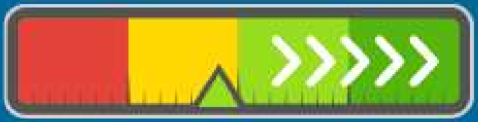 Lower risk

LOWER THE RISK OF INFECTING OTHERS

Prevent the spread of viruses
