# Supplementary material for: ‘Cough and sneeze into your elbow’: a field study testing the effects of persuasive messages on compliance with behavioral measures to prevent the spread of respiratory viruses
Source: Health Psychol Behav Med. 2026 Jan 20;14(1):2616931. doi: 10.1080/21642850.2026.2616931 (PMC12821337; doi:10.1080/21642850.2026.2616931)
Supplement: Supplementary_file_3_MANOVAs_descriptives_test_statistics.pdf [file RHPB_A_2616931_SM8639.pdf]

1. MANOVA 1 (Hypothesis 1)
    - IV: frequency of exposure
    - DVs: intention to comply at T1, attitude, social norm, moral norm, self-efficacy, response efficacy, risk perception
    - Covariate: behavior

Results

Table 1.

	Exposure frequency										
	0 times (*n* = 555)		1-2 times (*n* = 219)		3-4 times (*n* = 199)		5+ times (*n* = 125)				
	M	(SD)	M	(SD)	M	(SD)	M	(SD)	*F* (3, 1093)	*p*	η_p^2
Intention	3.51	1.23	3.79	1.20	3.66	1.29	3.66	1.21	1.98	.116	.005
Attitude	4.14	0.81	4.20	0.84	4.13	0.80	4.04	0.90	2.03	.108	.006
Social norm	**3.10**	0.92	**3.26**	0.83	**3.29**	0.83	3.20	0.89	2.77	.041*	.008
Moral norm	3.30	1.10	3.51	1.04	3.48	0.98	3.36	1.03	2.38	.068	.006
Self-efficacy	3.51	1.18	3.77	1.06	3.60	1.17	3.59	1.11	2.19	.088	.006
Response efficacy	4.36	0.79	4.41	0.74	4.42	0.71	4.37	0.72	0.62	.604	.002
Risk perception covid-19	**2.93**	0.61	**3.06**	0.55	**3.10**	0.62	3.05	0.62	5.04	.002**	.014
Risk perception flu	**2.86**	0.58	**2.99**	0.53	**2.99**	0.57	2.92	0.53	3.97	.008**	.011

Note: * $p < .05$, ** $p < .01$

A two-way MANOVA was conducted to test the effect of exposure frequency on intention and the various behavioral determinants.

There was a significant multivariate main effect of exposure frequency on the combined dependent variables, F(24, 3150) = 2.01, *p* = .002; Wilks' Λ = 0.957.

Univariate inspections further confirmed:

For social norm scores:
- Significant difference between 0 exposure and 1-2 exposures (*p* = .041)
- Significant difference between 0 exposure and 3-4 exposures (*p* = .012)

For risk perception Covid-19 scores:
- Significant difference between 0 exposure and 1-2 exposures (*p* = .010)
- Significant difference between 0 exposure and 3-4 exposures (*p* < .001)

For risk perception flu scores:
- Significant difference between 0 exposure and 1-2 exposures (*p* = .005)
- Significant difference between 0 exposure and 3-4 exposures (*p* = .007)

The significant differences are indicated in Table 1 in bold.

2. MANOVA 2 (RQ 2)
    - IV: frequency of exposure
    - DVs: intention to comply at T1, attitude, social norm, moral norm, self-efficacy, response efficacy, risk perception
    - Moderator: behavior

Results

A two-way MANOVA was conducted to test how the effect of exposure frequency on intention and the various behavioral determinants differs as a function of behavior type that was inquired in the questionnaire.

There was no significant interaction effect between exposure frequency and inquired behavior type on the combined dependent variables, $F(24, 3141) = 1.31$, $p = .142$; Wilks' $\Lambda = 0.972$.
There was, however, a significant multivariate main effect of inquired behavior type on the combined dependent variables, $F(8, 1083) = 111.61$, $p < .001$; Wilks' $\Lambda = 0.548$.

Univariate analyses of the effect of inquired behavior type on the individual dependent variables indicated a significant effect on all dependent variables except risk perception covid-19 (as derived from the 'tests of between subjects effects'-table). Specifically (as derived from the 'descriptives' table):
o Intention scores were significantly higher for coughing and sneezing than for staying at home when ill.
o Attitude scores were significantly higher for coughing and sneezing than for staying at home when ill.
o Social norm scores were significantly higher for coughing and sneezing than for staying at home when ill.
o Moral norm scores were significantly higher for coughing and sneezing than for staying at home when ill.
o Self-efficacy scores were significantly higher for coughing and sneezing than for staying at home when ill.
o Response efficacy scores were significantly higher for coughing and sneezing than for staying at home when ill.
o Risk perception flu scores were significantly higher for coughing and sneezing than for staying at home when ill.

Therefore, inquired behavior type will be included as a covariate in all subsequent analyses.

As we are mainly interested in how the scores on the multiple outcome variables differed for the two different behaviors (univariate comparisons rather than the combined outcome variables), and we used MANOVA to correct for multiple comparisons (as preferred over conducting several ANOVAs) rather than being interest in the effect on the combined outcomes, we still further inspected the univariate comparisons of this analysis.

Although there was no significant interaction effect between exposure frequency and inquired behavior type on the combined dependent variables, univariate analyses of the effect of exposure frequency * inquired behavior on the individual dependent variables indicated significant differences in 'Intention to comply' and 'self-efficacy' (derived from the 'Tests of Between-Subjects Effects' table). Univariate inspections (in the Pairwise Comparisons table, the second one (out of three) under the 'estimated marginal means') further confirmed:

For coughing and sneezing into the elbow:

- No significant differences between intention scores as a function of exposure frequency.
- No significant differences between self-efficacy scores as a function of exposure frequency.

For staying home when ill with symptoms:

- Significant difference in intention scores between 0 exposure and 1-2 exposures (p = .003), and between 1-2 exposures and 3-4 exposures (p = .006).
- Significant difference in self-efficacy scores between 0 exposure and 1-2 exposures (p < .001), between 1-2 exposures and 3-4 exposures (p = .008), and between 1-2 and 5+ times (p = .023).

All significant differences are indicated in Table 2.

Table 2. Means and standard deviations of inquired behavior type and univariate main and interaction effects on individual outcome measures.

	Exposure frequency												
	0 times (*n* = 555)		1-2 times (*n* = 219)		3-4 times (*n* = 199)		5+ times (*n* = 125)		Total (*n* = 1098)				
	M	(SD)	M	(SD)	M	(SD)	M	(SD)	M	(SD)			
Coughing and sneezing into elbow (n = 587)													
Intention	4.17	1.01	4.23	1.05	4.40	0.89	4.18	1.04	4.23 [1]	1.00			
Attitude	4.55	0.67	4.50	0.68	4.53	0.63	4.42	0.76	4.52 [2]	0.68			
Social norm	3.25	0.91	3.45	0.78	3.51	0.73	3.42	0.77	3.36 [3]	0.84			
Moral norm	3.48	1.02	3.69	1.00	3.70	0.92	3.53	0.99	3.57 [4]	1.00			
Self-efficacy	4.33	0.67	4.31	0.71	4.34	0.77	4.21	0.69	4.31 [5]	0.70			
Response efficacy	4.20	0.80	4.28	0.74	4.28	0.76	4.29	0.73	4.24 [6]	0.77			
Risk perception covid-19	2.93	0.64	3.07	0.53	3.17	0.61	3.07	0.61	3.02	0.61			
Risk perception flu	2.85	0.57	3.03	0.53	3.06	0.57	2.97	0.53	2.94 [7]	0.56			
Staying home when ill with symptoms (n= 511)													
Intention	**2.83**	1.05	**3.21**	1.15	**2.79**	1.14	2.92	1.06	2.90 [1]	1.09			
Attitude	3.72	0.72	3.81	0.86	3.66	0.71	3.50	0.79	3.70 [2]	0.76			
Social norm	2.94	0.90	3.01	0.82	3.02	0.87	2.90	0.96	2.97 [3]	0.89			
Moral norm	3.11	1.14	3.27	1.04	3.21	0.99	3.12	1.05	3.16 [4]	1.09			
Self-efficacy	**2.67**	0.97	**3.04**	1.02	**2.71**	0.94	**2.71**	0.99	2.75 [5]	0.98			
Response efficacy	4.53	0.75	4.57	0.70	4.59	0.61	4.48	0.70	4.55 [6]	0.71			
Risk perception covid-19	2.94	0.58	3.04	0.58	3.02	0.64	3.03	0.63	2.98	0.60			
Risk perception flu	2.87	0.58	2.94	0.54	2.90	0.56	2.86	0.54	2.89 [7]	0.57			

Behavior type inquired in questionnaire (coughing and sneezing or staying home)						F (1, 1090)	p	η_p^2
Intention						319.70	<.001**	0.227
Attitude						272.65	<.001**	0.200
Social norm						52.30	<.001**	0.046
Moral norm						33.21	<.001**	0.030
Self-efficacy						656.26	<.001**	0.376
Response efficacy						28.56	<.001**	0.026
Risk perception covid-19						1.67	.196	0.002
Risk perception flu						4.86	.028*	0.004
*Exposure frequency * Behavior type inquired in questionnaire*						F (3, 1090)	p	η_p^2
Intention						2.84	.037*	.008
Attitude						0.96	.412	.003
Social norm						0.94	.422	.003
Moral norm						0.16	.926	.000
Self-efficacy						2.94	.032*	.008
Response efficacy						0.33	.805	.001
Risk perception covid-19						0.95	.418	.003
Risk perception flu						1.45	.228	.004

Notes: (a) * $p < .05$, ** $p < .01$. *(b)* Significant differences in mean scores between inquired behavior types are indicated with a number in superscript (1-7) (comparisons within columns, vertically). *(c)* Significant differences within behavior type as a function of exposure frequency * inquired behavior type are indicated in bold (comparisons within rows, horizontally).

3. MANOVA 3 (RQ 3)
    - IV: frequency of exposure
    - DVs: intention to comply at T1, attitude, social norm, moral norm, self-efficacy, response efficacy, risk perception
    - Covariate: behavior
    - Moderator: educational level (participants' affiliated educational institution)

Results:

There was a significant multivariate main effect of affiliated educational institution on the combined dependent variables, $F(16, 2156) = 2.88$, $p < .001$; Wilks' $\Lambda = 0.959$.
There was no significant interaction effect between exposure frequency and participants' affiliated educational institution on the combined dependent variables, $F(48, 5308) = 1.16$, $p = .211$; Wilks' $\Lambda = 0.950$.

Univariate analyses of the effect of educational institution on the individual dependent variables indicated a significant main effect on social norm, moral norm and response efficacy. Specifically:

o Social norm scores were significantly higher for intermediate vocational education than for university of applied sciences;
o Social norm scores were significantly higher for intermediate vocational education than for university;
o Moral norm scores were significantly higher for university than for intermediate vocational education;
o Moral norm scores were significantly higher for university than for university of applied sciences;
o No significant differences between the educational institutions for response efficacy.

Table 3. Means and standard deviations stratified by affiliated educational institution and univariate main effects of educational institution on individual outcome measures.

	Exposure frequency									
	0 times (*n* = 555)		1-2 times (*n* = 219)		3-4 times (*n* = 199)		5+ times (*n* = 125)		Total (*n* = 1098)	
	M	(SD)	M	(SD)	M	(SD)	M	(SD)	M	(SD)
Intermediate vocational education (n = 63)										
Intention	4.47	0.87	4.13	1.04	4.25	0.95	4.50	0.73	4.34	0.90
Attitude	4.35	0.87	4.39	0.71	4.67	0.42	4.73	0.70	4.51	0.70
Social norm	3.38	0.77	3.59	1.04	4.03	0.70	3.84	0.86	3.66 [1,2]	0.88
Moral norm	3.14	1.03	3.26	0.99	3.20	0.59	3.47	1.15	3.27 [3]	0.98
Self-efficacy	4.42	0.79	4.42	0.87	4.20	0.67	4.47	0.69	4.40	0.76
Response efficacy	4.08	0.67	4.05	0.93	4.20	0.63	4.09	0.90	4.10	0.79
Risk perception covid-19	2.73	0.69	3.09	0.49	3.20	0.66	2.88	0.55	2.95	0.61
Risk perception flu	2.75	0.68	3.11	0.50	3.20	0.47	2.78	0.49	2.94	0.57
University of applied sciences (n = 211)										
Intention	3.35	1.33	3.74	1.31	3.73	1.24	3.53	1.21	3.57	1.29
Attitude	4.09	0.86	4.13	0.91	4.06	0.82	3.70	0.87	4.03	0.87
Social norm	3.10	0.90	3.22	0.80	3.13	0.65	2.91	0.91	3.11 [1]	0.82
Moral norm	3.19	1.10	3.33	1.13	3.37	0.99	3.25	1.07	3.28 [4]	1.07
Self-efficacy	3.69	0.99	3.68	1.05	3.52	1.08	3.26	1.05	3.57	1.04
Response efficacy	4.20	0.83	4.36	0.81	4.35	0.86	4.40	0.64	4.31	0.80
Risk perception covid-19	2.91	0.64	3.03	0.48	3.14	0.56	3.04	0.69	3.02	0.60
Risk perception flu	2.76	0.57	2.96	0.44	2.96	0.53	2.93	0.59	2.89	0.54

University (n = 824)													
Intention	3.50	1.21	3.76	1.19	3.59	1.33	3.53	1.24	3.57	1.23			
Attitude	4.14	0.80	4.20	0.83	4.12	0.80	4.05	0.89	4.14	0.81			
Social norm	3.08	0.92	3.24	0.80	3.30	0.88	3.20	0.82	3.16 ^{2}	0.89			
Moral norm	3.32	1.10	3.60	1.01	3.55	1.00	3.38	0.99	3.41 ^{3,4}	1.06			
Self-efficacy	3.45	1.20	3.71	1.06	3.59	1.23	3.55	1.12	3.53	1.18			
Response efficacy	4.40	0.79	4.46	0.68	4.47	0.64	4.42	0.71	4.43	0.74			
Risk perception covid-19	2.94	0.60	3.06	0.58	3.08	0.65	3.10	0.60	3.00	0.61			
Risk perception flu	2.88	0.57	2.99	0.56	2.99	0.60	2.95	0.51	2.92	0.57			
Educational institution											F (2, 1085)	p	η_p^2
Intention											0.29	.746	0.001
Attitude											2.37	.094	0.004
Social norm											5.86	.003**	0.011
Moral norm											5.13	.006**	0.009
Self-efficacy											0.19	.823	0.000
Response efficacy											3.04	.048*	0.006
Risk perception covid-19											0.61	.542	0.001
Risk perception flu											0.53	.588	0.001

Notes: *(a)* * $p < .05$, ** $p < .01$. *(b)* Significant differences in mean scores between educational institutions are indicated with a number in superscript (1-4) (comparisons within columns, vertically).

4. MANOVA 4 (RQ 1)
    - IV: frequency of exposure
    - DVs: intention to comply at T1, attitude, social norm, moral norm, self-efficacy, response efficacy, risk perception
    - Covariate: behavior
    - Moderator: role (student vs. employee)

Results:

There was no significant interaction effect between exposure frequency and role (student or employee) on the combined dependent variables, $F(24, 3139) = 0.548$, $p = .963$; Wilks' $\Lambda = 0.988$. There was a significant multivariate main effect of role (student or employee) on the combined dependent variables, $F(8, 1082) = 5.167$, $p < .001$; Wilks' $\Lambda = 0.963$.

Univariate analyses of the effect of role on the individual dependent variables indicated a significant main effect on intention, social norm, and self-efficacy. Specifically:

o Intention scores were significantly higher for employees than for students;
o Social norm scores were significantly higher for students than for employees;
o Self-efficacy scores were significantly higher for employees than for students.

Table 4. Means and standard deviations stratified by role (student or employee) and univariate main effects of role on individual outcome measures.

	Total (*n* = 1098)				
	M	(SD)			
Student (n = 751)					
Intention	3.55 [1]	1.27			
Attitude	4.12	0.83			
Social norm	3.20 [2]	0.92			
Moral norm	3.33	1.08			
Self-efficacy	3.55 [3]	1.22			
Response efficacy	4.36	0.77			
Risk perception covid-19	2.97	0.60			
Risk perception flu	2.93	0.58			
Employee (n = 347)					
Intention	3.75 [1]	1.17			
Attitude	4.18	0.80			
Social norm	3.13 [2]	0.79			
Moral norm	3.48	1.01			
Self-efficacy	3.67 [3]	0.99			

Response efficacy	4.43	0.73			
Risk perception covid-19	3.07	0.60			
Risk perception flu	2.90	0.54			
Role (student or employee)			*F* (1, 1089)	*p*	η_p^2
Intention			8.71	.003*	0.008
Attitude			1.98	.160	0.002
Social norm			4.32	.038*	0.004
Moral norm			2.50	.114	0.002
Self-efficacy			5.09	.024*	0.005
Response efficacy			0.17	.678	0.000
Risk perception covid-19			3.33	.068	0.003
Risk perception flu			0.67	.414	0.001

Notes: *(a)* * p < .05, ** p < .01. *(b)* Significant differences in mean scores between students and employees are indicated with a number in superscript (1-3) (comparisons within columns, vertically).

5. MANOVA 5 (RQ 5)
    - IV: frequency of exposure
    - DVs: intention to comply at T1, attitude, social norm, moral norm, self-efficacy, response efficacy, risk perception
    - Covariate: behavior
    - Moderator: participation at T0

There was no significant interaction effect between exposure frequency and participation in T0 (yes or no) on the combined dependent variables, $F(24, 3139) = 0.740$, $p = .813$; Wilks' $\Lambda = 0.984$.
There was a significant multivariate main effect of participation in T0 (yes or no) on the combined dependent variables, $F(8, 1082) = 2.638$, $p = .007$; Wilks' $\Lambda = 0.981$.

Univariate analyses of the effect of participation at T0 (yes or no) on the individual dependent variables indicated a significant main effect on moral norm. Specifically:

o Moral norm scores were significantly higher for participants who participated in T0, than for participants who did not participate in T0.

Table 5. Means and standard deviations stratified by participation in T0 (yes or no) and univariate main effects of participation in T0 on individual outcome measures.

	Total (*n* = 1098)				
	M	(SD)			
Participated in T0 (n = 526)					
Intention	3.49	1.24			
Attitude	4.08	0.80			
Social norm	3.17	0.91			
Moral norm	3.40 [1]	1.07			
Self-efficacy	3.46	1.19			
Response efficacy	4.39	0.76			
Risk perception covid-19	3.00	0.61			
Risk perception flu	2.94	0.57			
Did not participate in T0 (n = 572)					
Intention	3.72	1.22			
Attitude	4.20	0.84			
Social norm	3.18	0.86			
Moral norm	3.36 [1]	1.06			
Self-efficacy	3.70	1.10			
Response efficacy	4.38	0.76			
Risk perception covid-19	3.00	0.60			
Risk perception flu	2.90	0.56			

Participation in T0 (yes or no)		*F* (1, 1089)	*p*	η_p^2
Intention		2.14	.144	0.002
Attitude		1.43	.232	0.001
Social norm		0.52	.469	0.000
Moral norm		4.40	.036*	0.004
Self-efficacy		3.09	.079	0.003
Response efficacy		0.15	.704	0.000
Risk perception covid-19		0.01	.917	0.000
Risk perception flu		1.82	.178	0.002

Notes: *(a)* * p < .05, ** p < .01. *(b)* Significant differences in mean scores between participants who did and did not participate in T0 are indicated with a number in superscript (1) (comparison within columns, vertically).

6. MANOVA 6 (RQ 3)
    - IV: frequency of exposure
    - DVs: intention to comply at T1, attitude, social norm, moral norm, self-efficacy, response efficacy, risk perception
    - Covariate: behavior
    - Moderator: gender

There was no significant interaction effect between exposure frequency and gender on the combined dependent variables, $F(24, 3060) = 1.198$, $p = .231$; Wilks' $\Lambda = 0.973$.
There was a significant multivariate main effect of gender on the combined dependent variables, $F(8, 1055) = 5.938$, $p < .001$; Wilks' $\Lambda = 0.957$.

Univariate analyses of the effect of gender (male or female) on the individual dependent variables indicated significant main and interaction effects on individual dependent variables. Specifically:

Univariate main effects:
o Intention, attitude, moral norm, risk perception covid-19, and risk perception flu scores were significantly higher for females than for males.

Univariate interaction effects:
o For males, intention scores were significantly **lower** for males who were exposed to messages 5+ times ($M = 3.03$, $SD = 1.26$) than for males who were exposed 0 times ($M = 3.44$, $SD = 1.37$; $p = .008$), for males who were exposed 1-2 times ($M = 3.69$, $SD = 1.20$; $p <.001$), and for males who were exposed 3-4 times ($M = 3.41$, $SD = 1.48$; $p = .024$).

o For females, intention scores were significantly **higher** for females who were exposed to messages 5+ times ($M = 3.86$, $SD = 1.12$) than for females who were exposed 0 times ($M = 3.53$, $SD = 1.19$; $p = .042$).

Table 6. Means and standard deviations stratified by gender (male or female) and exposure frequency, and univariate main and interaction effects of gender on individual outcome measures.

	Exposure frequency												
	0 times (*n* = 545)		1-2 times (*n* = 209)		3-4 times (*n* = 194)		5+ times (*n* = 123)		Total (*n* = 1071)				
	M	(SD)	M	(SD)	M	(SD)	M	(SD)	M	(SD)			
Males (n = 257)													
Intention	**3.44**	1.37	**3.69**	1.20	**3.41**	1.48	**3.03**	1.26	3.44 [1]	1.35			
Attitude	4.07	0.87	4.13	0.86	4.03	0.81	3.70	0.94	4.03 [2]	0.87			
Social norm	3.09	0.87	3.33	0.81	3.29	097	3.11	0.81	3.17	0.87			
Moral norm	3.05	1.16	3.43	1.07	3.29	1.01	3.17	1.04	3.18 [3]	1.11			
Self-efficacy	3.53	1.14	3.75	1.05	3.55	1.15	3.50	0.90	3.58	1.09			
Response efficacy	4.37	0.74	4.35	0.82	4.43	0.78	4.12	0.93	4.34	0.79			
Risk perception covid-19	2.72	0.61	2.91	0.64	2.85	0.68	2.92	0.53	2.80 [4]	0.62			
Risk perception flu	2.65	0.63	2.85	0.58	2.72	0.55	2.76	0.49	2.71 [5]	0.59			
Females (n = 814)													
Intention	**3.53**	1.19	3.86	1.20	3.72	1.24	**3.86**	1.12	3.66 [1]	1.20			
Attitude	4.16	0.79	4.26	0.81	4.15	0.80	4.15	0.85	4.17 [2]	0.80			
Social norm	3.10	0.93	3.25	0.84	3.30	0.78	3.25	0.92	3.18	0.89			
Moral norm	3.36	1.08	3.52	1.03	3.52	0.98	3.42	1.02	3.43 [3]	1.04			
Self-efficacy	3.50	1.19	3.81	1.02	3.60	1.17	3.60	1.18	3.59	1.16			
Response efficacy	4.35	0.81	4.41	0.72	4.43	0.69	4.46	0.61	4.39	0.75			
Risk perception covid-19	3.00	0.60	3.11	0.49	3.16	0.60	3.09	0.64	3.06 [4]	0.59			
Risk perception flu	2.93	0.54	3.04	0.51	3.05	0.56	2.98	0.54	2.98 [5]	0.54			

Gender (male or female)						*F* (1, 1062)	*p*	η_p^2
Intention						11.21	<.001**	0.010
Attitude						7.41	.007*	0.007
Social norm						0.00	.983	0.000
Moral norm						5.77	.016*	0.005
Self-efficacy						0.12	.732	0.000
Response efficacy						3.15	.076	0.003
Risk perception covid-19						24.88	<.001**	0.023
Risk perception flu						30.79	<.001**	0.028
*Gender * Exposure frequency*						*F* (3, 1062)	*p*	η_p^2
Intention						4.34	.005*	0.012
Attitude						2.47	.061	0.007
Social norm						0.61	.607	0.002
Moral norm						0.75	.523	0.002
Self-efficacy						0.80	.492	0.002
Response efficacy						1.48	.218	0.004
Risk perception covid-19						0.42	.740	0.001
Risk perception flu						0.54	.653	0.002

Notes: *(a)* * p < .05, ** p < .01. *(b)* Significant differences in mean scores between males and females are indicated with a number in superscript (1-5) (comparisons within columns, vertically). *(c)* Significant differences in mean scores within a gender as a function of exposure frequency * gender are indicated in bold (comparisons within rows, horizontally).

7. MANOVA 7 (RQ 3)

- IV: frequency of exposure
- DVs: intention to comply at T1, attitude, social norm, moral norm, self-efficacy, response efficacy, risk perception
- Covariate: behavior
- Moderator: age

There was no significant interaction effect between exposure frequency and age group (16-24; 25-39; 40-59; 60+ years) on the combined dependent variables, $F(72, 6540) = 0.949$, $p = .600$; Wilks' Λ = 0.939.
There was a significant multivariate main effect of age group (16-24; 25-39; 40-59; 60+ years) on the combined dependent variables, $F(24, 3116) = 2.721$, $p < .001$; Wilks' Λ = 0.942.

Univariate analyses of the effect of age group on the individual dependent variables indicated a significant main effect. Specifically:

o Social norm scores were significantly higher for participants aged 16-24 years (M = 3.23, SD = 0.92), than for participants aged 25-39 years (M = 3.03, SD = 0.82, p = .003).

Table 7. Means and standard deviations stratified by age group and univariate main effects of age group on individual outcome measures.

	Total (*n* = 1098)				
	M	(SD)			
16-24 years (n = 692)					
Intention	3.55	1.26			
Attitude	4.12	0.83			
Social norm	3.23[1]	0.92			
Moral norm	3.33	1.06			
Self-efficacy	3.56	1.22			
Response efficacy	4.35	0.78			
Risk perception covid-19	2.97	0.59			
Risk perception flu	2.93	0.57			
25-39 years (n = 226)					
Intention	3.68	1.22			
Attitude	4.21	0.80			
Social norm	3.03[1]	0.82			
Moral norm	3.42	1.09			
Self-efficacy	3.66	1.05			
Response efficacy	4.49	0.67			
Risk perception covid-19	3.04	0.63			
Risk perception flu	2.88	0.57			

40-59 years (n = 153)					
Intention	3.77	1.15			
Attitude	4.13	0.83			
Social norm	3.17	0.82			
Moral norm	3.52	1.03			
Self-efficacy	3.58	1.00			
Response efficacy	4.38	0.80			
Risk perception covid-19	3.11	0.61			
Risk perception flu	2.92	0.54			
60+ years					
(n = 27)					
Intention	3.78	1.24			
Attitude	4.15	0.84			
Social norm	3.16	0.64			
Moral norm	3.61	0.92			
Self-efficacy	3.72	0.92			
Response efficacy	4.35	0.77			
Risk perception covid-19	3.07	0.63			
Risk perception flu	2.81	0.53			
Age group (16-24; 25-39; 40-59; 60+ years)			*F* (3, 1081)	*p*	η_p^2
Intention			2.27	.079	0.006
Attitude			1.56	.198	0.004
Social norm			3.23	.022*	0.009
Moral norm			0.84	.473	0.002
Self-efficacy			2.30	.076	0.006
Response efficacy			0.93	.472	0.003
Risk perception covid-19			0.81	.489	0.002
Risk perception flu			0.56	.641	0.002

Notes: *(a)* * p < .05, ** p < .01. *(b)* Significant differences in mean scores between participants of different age groups are indicated with a number in superscript (1) (comparison within columns, vertically).

8. MANOVA 8 (RQ 3)
    - IV: frequency of exposure
    - DVs: intention to comply at T1, attitude, social norm, moral norm, self-efficacy, response efficacy, risk perception
    - Covariate: behavior
    - Moderator: ethnic background

There was no significant interaction effect between exposure frequency and ethnic background (Netherlands; Non-western migration background; Western migration background) on the combined dependent variables, $F(48, 5289) = 1.288$, $p = .088$; Wilks' $\Lambda = 0.944$.
There was a significant multivariate main effect of ethnic background (Netherlands; Non-western migration background; Western migration background) on the combined dependent variables, $F(16, 2148) = 6.267$, $p < .001$; Wilks' $\Lambda = 0.913$.

Univariate analyses of the effect of ethnic background on the individual dependent variables indicated significant main and interaction effects on individual dependent variables. Specifically:

Univariate main effects:
o Social norm scores were significantly higher for participants with ethnic background 'Netherlands' (*M* = 3.09, *SD* = 0.89) than for participants with a Western migration background (*M* = 3.27, *SD* = 0.86, *p* = .001) and for participants with a Non-western migration background (*M* = 3.27, *SD* = 0.89, *p* = .023).

o Moral norm scores were significantly higher for participants for participants with a Western migration background (*M* = 3.56, *SD* = 1.07, *p* < .001) and for participants with a Non-western migration background (*M* = 3.43, *SD* = 1.06, *p* = .033), compared to participants with ethnic background 'Netherlands' (*M* = 3.25, *SD* = 1.04).

o Risk perception covid-19 scores were significantly higher for participants with a Non-Western migration background (*M* = 3.20, *SD* = 0.64) than for participants with ethnic background 'Netherlands' (*M* = 2.92, *SD* = 0.56, *p* < .001) and for participants with a Western migration background (*M* = 3.01, *SD* = 0.62, *p* < .001).

o Risk perception flu scores were significantly higher for participants with a Non-Western migration background (*M* = 3.12, *SD* = 0.58) than for participants with ethnic background 'Netherlands' (*M* = 2.84, *SD* = 0.51, *p* < .001) and for participants with a Western migration background (*M* = 2.93, *SD* = 0.61, *p* < .001). In addition, risk perception flu scores were significantly higher for participants with a Western migration background than for participants with ethnic background 'Netherlands' (*p* = .049).

Univariate interaction effects:

o For participants with ethnic background 'Netherlands', risk perception covid-19 scores were significantly higher for participants who were exposed to messages 1-2 times (*M* = 3.02, *SD* = 0.50; *p* = .006), and for participants who were exposed to messages 3-4 times (*M* = 3.00, *SD* = 0.57, *p* = .009), than for participants who were exposed to messages 0 times (*M* = 2.84, *SD* = 0.58).

o For participants with ethnic background 'Netherlands', risk perception flu scores were significantly higher for participants who were exposed to messages 1-2 times (*M* = 2.92, *SD* = 0.45; *p* = .024), and participants who were exposed to messages 3-4 times (*M* = 2.91, *SD* = 0.51; *p* = .028), than for participants who were exposed to messages 0 times (*M* = 2.78, *SD* = 0.54).

o For participants with a Non-western migration background, risk perception covid-19 scores were significantly higher for participants who were exposed to messages 3-4 times (*M* = 3.53, *SD* = 0.60, $p < .001$) and 5+ times (*M* = 3.45, *SD* = 0.70, $p = .002$) , than participants who were exposed 0 times (*M* = 3.04, *SD* = 0.61). In addition, risk perception covid-19 scores were significantly higher for participants who were exposed to messages 3-4 times ($p = .003$) and 5+ times ($p = .031$) than for participants who were exposed to messages 1-2 times.

o For participants with a Non-western migration background, risk perception flu scores were significantly higher for participants who were exposed to messages 3-4 times (*M* = 3.34, *SD* = 0.64; $p < .001$) and 5+ times (*M* = 3.33, *SD* = 0.54; $p = .003$), than for participants who were exposed to messages 0 times (*M* = 2.97, *SD* = 0.54).

o For participants with a Western migration background, there were no significant differences in risk perception covid-19 scores nor in risk perception flu scores as a function of ethnic background * exposure frequency.

Table 8. Means and standard deviations stratified by ethnic background (Netherlands; Non-western migration background; Western migration background) and exposure frequency, and univariate main and interaction effects of ethnic background on individual outcome measures.

	Exposure frequency												
	0 times (*n* = 553)		1-2 times (*n* = 218)		3-4 times (*n* = 199)		5+ times (*n* = 124)		Total (*n* = 1094)				
	M	(SD)	M	(SD)	M	(SD)	M	(SD)	M	(SD)			
Netherlands (n = 565)													
Intention	3.52	1.29	3.75	1.25	3.64	1.32	3.72	1.24	3.62	1.28			
Attitude	4.16	0.86	4.22	0.87	4.13	0.82	4.00	0.91	4.14	0.86			
Social norm	3.01	0.90	3.16	0.90	3.14	0.85	3.18	0.91	3.09 [1,2]	0.89			
Moral norm	3.16	1.07	3.43	1.03	3.33	0.98	3.18	1.04	3.25 [3,4]	1.04			
Self-efficacy	3.56	1.16	3.78	1.13	3.54	1.18	3.54	1.15	3.60	1.16			
Response efficacy	4.32	0.75	4.43	0.70	4.42	0.73	4.34	0.77	4.37	0.74			
Risk perception covid-19	**2.84**	0.58	**3.02**	0.50	**3.00**	0.57	2.90	0.55	2.92 [5]	0.56			
Risk perception flu	**2.78**	0.54	**2.92**	0.45	**2.91**	0.51	2.76	0.46	2.84 [7,8]	0.51			
Non-western migration background (n = 214)													
Intention	3.50	1.11	3.67	1.27	3.43	1.30	3.78	1.05	3.56	1.17			
Attitude	4.18	0.73	4.05	0.88	3.99	0.83	4.11	0.93	4.11	0.80			
Social norm	3.20	0.92	3.29	0.80	3.45	0.81	3.22	1.00	3.27 [1]	0.89			
Moral norm	3.39	1.05	3.35	1.21	3.59	0.89	3.52	1.09	3.43 [3]	1.06			
Self-efficacy	3.57	1.16	3.72	0.97	3.68	1.14	3.74	1.08	3.65	1.10			
Response efficacy	4.35	0.87	4.11	0.93	4.41	0.67	4.48	0.51	4.32	0.82			
Risk perception covid-19	**3.04**	0.61	**3.14**	0.56	**3.53**	0.60	**3.45**	0.70	3.20 [5,6]	0.64			
Risk perception flu	**2.97**	0.54	3.16	0.57	**3.34**	0.64	**3.33**	0.54	3.12 [7,9]	0.58			

Western migration background (n = 315)													
Intention	3.49	1.22	3.99	1.05	3.92	1.19	3.31	1.27	3.62	1.21			
Attitude	4.10	0.79	4.30	0.72	4.26	0.68	4.04	0.87	4.15	0.77			
Social norm	3.14	0.94	3.46	0.65	3.55	0.71	3.25	0.73	3.27 [2]	0.86			
Moral norm	3.42	1.15	3.79	0.87	3.78	1.00	3.67	0.86	3.56 [4]	1.07			
Self-efficacy	3.40	1.21	3.77	1.01	3.69	1.20	3.52	1.03	3.52	1.17			
Response efficacy	4.43	0.81	4.58	0.56	4.45	0.71	4.35	0.77	4.45	0.75			
Risk perception covid-19	3.00	0.62	3.05	0.62	2.98	0.64	3.07	0.54	3.01 [6]	0.62			
Risk perception flu	2.91	0.63	2.98	0.63	2.88	0.56	2.97	0.52	2.93 [8,9]	0.61			
Ethnic background (Netherlands, Non-western, Western)											F (2, 1081)	p	η_p^2
Intention											0.24	.784	0.000
Attitude											0.83	.434	0.002
Social norm											6.20	.002**	0.011
Moral norm											10.57	<.001**	0.019
Self-efficacy											1.25	.287	0.002
Response efficacy											1.06	.347	0.002
Risk perception covid-19											21.30	<.001**	0.038
Risk perception flu											25.22	<.001**	0.045
*Ethnic background * Exposure frequency*											F (6, 1081)	p	η_p^2
Intention											1.86	.085	0.010
Attitude											1.05	.391	0.006

Social norm						0.58	.748	0.003
Moral norm						0.70	.651	0.004
Self-efficacy						0.51	.802	0.003
Response efficacy						1.35	.233	0.007
Risk perception covid-19						3.10	.005**	0.017
Risk perception flu						2.42	.025*	0.013

Notes: *(a)* * p < .05, ** p < .01. *(b)* Significant differences in mean scores between males and females are indicated with a number in superscript (1-9) (comparisons within columns, vertically). *(c)* Significant differences in mean scores within a gender as a function of exposure frequency * gender are indicated in bold (comparisons within rows, horizontally).

9. MANOVA 9 (RQ 4)

- IV: frequency of exposure
- DVs: intention to comply at T1, attitude, social norm, moral norm, self-efficacy, response efficacy, risk perception
- Covariate: behavior
- Moderator: knowledge of viruses

There was no significant interaction effect between exposure frequency and knowledge of respiratory viruses and preventive measures (low or high) on the combined dependent variables, $F(24, 3139) = 0.900$, $p = .604$; Wilks' $\Lambda = 0.980$.
There was a significant multivariate main effect of knowledge of respiratory viruses and preventive measures (low or high) on the combined dependent variables, $F(8, 1082) = 8.760$, $p < .001$; Wilks' $\Lambda = 0.939$.

Univariate analyses of the effect of knowledge of respiratory viruses and preventive measures (low or high) on the individual dependent variables indicated a significant main effect on intention, attitude, moral norm, and response efficacy. Specifically:

o Intention, attitude, moral norm, and response efficacy scores were significantly higher for participants with high levels of knowledge than for participants with low levels of knowledge of respiratory viruses and preventive measures (Table 9).

Table 9. Means and standard deviations stratified by knowledge of respiratory viruses and preventive measures (low or high) and univariate main effects of knowledge on respiratory viruses on individual outcome measures.

	Total (*n* = 1098)				
	M	(SD)			
Knowledge low (n = 300)					
Intention	3.55 [1]	1.26			
Attitude	4.03 [2]	0.88			
Social norm	3.22	0.90			
Moral norm	3.21 [3]	1.07			
Self-efficacy	3.72	1.08			
Response efficacy	4.07 [4]	0.87			
Risk perception covid-19	2.97	0.66			
Risk perception flu	2.91	0.61			
Knowledge high (n = 798)					
Intention	3.63 [1]	1.23			
Attitude	4.18 [2]	0.80			
Social norm	3.16	0.87			
Moral norm	3.44 [3]	1.05			

Self-efficacy	3.54	1.17			
Response efficacy	4.50 [4]	0.68			
Risk perception covid-19	3.02	0.58			
Risk perception flu	2.92	0.55			
Knowledge of respiratory viruses and preventive measures (low or high)			F (1, 1089)	p	η_p^2
Intention			7.72	.006**	0.007
Attitude			18.11	<.001**	0.016
Social norm			1.28	.259	0.001
Moral norm			6.78	.009**	0.006
Self-efficacy			0.10	.754	0.000
Response efficacy			42.47	<.001**	0.038
Risk perception covid-19			0.66	.417	0.001
Risk perception flu			0.15	.695	0.000

Notes: *(a)* * p < .05, ** p < .01. *(b)* Significant differences in mean scores between participants with low or high levels of knowledge are indicated with a number in superscript (1-4) (comparison within columns, vertically).

10. MANOVA 10 (RQ 4)

- o IV: frequency of exposure
- o DVs: intention to comply at T1, attitude, social norm, moral norm, self-efficacy, response efficacy, risk perception
- o Covariate: behavior
- o Moderator: trust in government

There was no significant interaction effect between exposure frequency and trust in government (low or high) on the combined dependent variables, $F(24, 3139) = 0.929$, $p = .562$; Wilks' $\Lambda = 0.980$.
There was a significant multivariate main effect of trust in government (low or high) on the combined dependent variables, $F(8, 1082) = 2.396$, $p = .015$; Wilks' $\Lambda = 0.983$.

Univariate analyses of the effect of trust in government (low or high) on the individual dependent variables indicated a significant main effect on response efficacy. Specifically:

o Response efficacy scores were significantly higher for participants with high trust in government than for participants with low trust in government (Table 10).

Table 10. Means and standard deviations stratified by trust in government (low or high) and univariate main effects of trust in government on individual outcome measures.

	Total (*n* = 1098)				
	M	(SD)			
Trust in government low (n = 611)					
Intention	3.57	1.23			
Attitude	4.10	0.82			
Social norm	3.18	0.88			
Moral norm	3.31	1.09			
Self-efficacy	3.52	1.20			
Response efficacy	4.29 [1]	0.84			
Risk perception covid-19	2.97	0.63			
Risk perception flu	2.91	0.58			
Trust in government high (n = 487)					
Intention	3.66	1.24			
Attitude	4.19	0.82			
Social norm	3.17	0.89			
Moral norm	3.47	1.03			
Self-efficacy	3.67	1.08			
Response efficacy	4.50 [1]	0.63			
Risk perception covid-19	3.04	0.57			
Risk perception flu	2.93	0.54			

Trust in government (low or high)		F (1, 1089)	p	η_p^2
Intention		0.20	.657	0.000
Attitude		0.43	.510	0.000
Social norm		1.03	.311	0.001
Moral norm		2.18	.140	0.002
Self-efficacy		1.13	.289	0.001
Response efficacy		13.12	<.001**	0.012
Risk perception covid-19		0.90	.344	0.001
Risk perception flu		0.05	.820	0.000

Notes: *(a)* * p < .05, ** p < .01. *(b)* Significant differences in mean scores between participants with low or high trust in government are indicated with a number in superscript (1) (comparison within columns, vertically).

11. MANOVA 11 (RQ 4)

- IV: frequency of exposure
- DVs: intention to comply at T1, attitude, social norm, moral norm, self-efficacy, response efficacy, risk perception
- Covariate: behavior
- Moderator: prosocial orientation

There was no significant interaction effect between exposure frequency and prosocial orientation (self or others) on the combined dependent variables, $F(24, 3139) = 1.315$, $p = .139$; Wilks' $\Lambda = 0.971$. There was a significant multivariate main effect of prosocial orientation (self or others) on the combined dependent variables, $F(8, 1082) = 4.619$, $p < .001$; Wilks' $\Lambda = 0.967$.

Univariate analyses of the effect of prosocial orientation (self or others) on the individual dependent variables indicated a significant main effect on response efficacy, risk perception covid-19, and risk perception flu (Table 11). Specifically:

o Response efficacy scores were significantly higher for participants who were predominantly <u>other-oriented</u> than for participants who were predominantly self-oriented.
o Risk perception covid-19 scores were significantly higher for participants who were predominantly <u>self-oriented</u> than for participants who were predominantly other-oriented.
o Risk perception flu scores were significantly higher for participants who were predominantly <u>self-oriented</u> than for participants who were predominantly other-oriented.

Table 11. Means and standard deviations stratified by prosocial orientation (self or others) and univariate main effects of prosocial orientation on individual outcome measures.

	Total (*n* = 1098)				
	M	(SD)			
Self-oriented (n = 426)					
Intention	3.60	1.27			
Attitude	4.11	0.83			
Social norm	3.22	0.85			
Moral norm	3.37	1.10			
Self-efficacy	3.59	1.15			
Response efficacy	4.30 [1]	0.81			
Risk perception covid-19	3.10 [2]	0.66			
Risk perception flu	3.00 [3]	0.61			
Other-oriented (n = 672)					
Intention	3.62	1.22			
Attitude	4.16	0.82			
Social norm	3.15	0.90			
Moral norm	3.38	1.04			

Self-efficacy	3.58	1.15			
Response efficacy	4.44[1]	0.72			
Risk perception covid-19	2.94[2]	0.56			
Risk perception flu	2.87[3]	0.53			
Prosocial orientation (self or others)			*F* (1, 1089)	*p*	η_p^2
Intention			0.82	.365	0.001
Attitude			1.22	.269	0.001
Social norm			1.63	.202	0.001
Moral norm			0.06	.814	0.000
Self-efficacy			0.04	.844	0.000
Response efficacy			7.25	.007**	0.007
Risk perception covid-19			15.00	<.001**	0.014
Risk perception flu			15.88	<.001**	0.014

Notes: *(a)* * p < .05, ** p < .01. *(b)* Significant differences in mean scores between participants who are predominantly self- or other-oriented are indicated with a number in superscript (1-3) (comparison within columns, vertically).

12. MANOVA 12 (RQ 4)

- IV: frequency of exposure
- DVs: intention to comply at T1, attitude, social norm, moral norm, self-efficacy, response efficacy, risk perception
- Covariate: behavior
- Moderator: perceived health status

There was no significant interaction effect between exposure frequency and perceived health status (bad or good) on the combined dependent variables, $F(24, 3139) = 0.919$, $p = .576$; Wilks' $\Lambda = 0.980$. There was a significant multivariate main effect of perceived health status (bad or good) on the combined dependent variables, $F(8, 1082) = 8.086$, $p < .001$; Wilks' $\Lambda = 0.944$.

Univariate analyses of the effect of perceived health status (bad or good) on the individual dependent variables indicated a significant main effect on risk perception covid-19 and risk perception flu (Table 12). Specifically:

o Risk perception covid-19 scores were significantly higher for participants with a bad rather than good perceived health status.
o Risk perception flu scores were significantly higher for participants with a bad rather than good perceived health status.

Table 12. Means and standard deviations stratified by perceived health status (bad or good) and univariate main effects of perceived health status on individual outcome measures.

	Total (*n* = 1098)				
	M	(SD)			
Bad perceived health status (n = 271)					
Intention	3.75	1.21			
Attitude	4.22	0.79			
Social norm	3.25	0.87			
Moral norm	3.47	1.05			
Self-efficacy	3.69	1.14			
Response efficacy	4.30	0.81			
Risk perception covid-19	3.24 [1]	0.66			
Risk perception flu	3.18 [2]	0.60			
Good perceived health status (n = 827)					
Intention	3.57	1.25			
Attitude	4.12	0.83			
Social norm	3.15	0.89			
Moral norm	3.35	1.06			
Self-efficacy	3.55	1.15			

Response efficacy	4.41	0.74			
Risk perception covid-19	2.92 [1]	0.56			
Risk perception flu	2.83 [2]	0.53			
Perceived health status (bad or good)			*F* (1, 1089)	*p*	η_p^2
Intention			1.89	.169	0.002
Attitude			0.37	.544	0.000
Social norm			0.38	.537	0.000
Moral norm			0.94	.331	0.001
Self-efficacy			0.45	.502	0.000
Response efficacy			0.54	.463	0.000
Risk perception covid-19			41.53	<.001**	0.037
Risk perception flu			54.71	<.001**	0.048

Notes: *(a)* * p < .05, ** p < .01. *(b)* Significant differences in mean scores between participants who perceived their health status as bad or as good are indicated with a number in superscript (1-2) (comparison within columns, vertically).
