# Supplementary material for: ‘Cough and sneeze into your elbow’: a field study testing the effects of persuasive messages on compliance with behavioral measures to prevent the spread of respiratory viruses
Source: Health Psychol Behav Med. 2026 Jan 20;14(1):2616931. doi: 10.1080/21642850.2026.2616931 (PMC12821337; doi:10.1080/21642850.2026.2616931)
Supplement: Supplementary_file_2_Overview_measures.pdf [file RHPB_A_2616931_SM8640.pdf]

Supplementary file I. Overview of measures, answer scales, justifications, and example items.

**Demographic characteristics**	**Number of items and type of answer scale**	**Adapted from**	**Example item(s)**
Age in years	1 item, numerical	n/a	What is your age? [0-100]
Gender	1 item, categorical	n/a	Are you: [Man, Woman, Other, Prefer not to say]
Educational institution	1 item, categorical	n/a	At which educational institution are you enrolled as a student / affiliated with as an employee? [Intermediate vocational education, University of applied sciences, Research University A, Research University B]
Role	1 item, categorical	n/a	Are you a student or employee at this educational institution? [Student, Employee, Neither]
Ethnic background	3 items, categorical	Based on the categories indicating non, a western or non-western migration background from Statistics Netherlands (CBS, 2022)	In which country were you born? And your parents/caretakers? (Me, Parent/Caretaker 1, Parent/Caretaker 2)<br>[Netherlands; Netherlands Antilles or Aruba; Indonesia; Morocco; Turkey; Surinam; Australia; Somewhere else within Europe or North America; Somewhere else outside of Europe or North America; Not applicable]
**Exposure recall and evaluation***	**Number of items and answer scales**	**Adapted from**	**Example item(s) and answer options**

Which information*	1 item, categorical	n/a	In the past weeks, did you see any information via your educational institution about prevention of virus spreading (such as coughing and sneezing in your elbow, or staying at home when you are ill with respiratory infection symptoms, such as coughing, sneezing, sore throat, and a runny or stuffy nose)? Please indicate which information you have seen. You can indicate multiple answers.<br>[Poster 1; Poster 2; Poster 3; Poster 4; Poster 5; Poster 6; I have seen information, but don't remember which; I did not see any of the above information]
Where*	1 item, categorical	n/a	Where did you see the information? You can indicate multiple answers.<br>[Student information website; Website for employees; App of my educational institution; Poster(s) in the elevator; Information screens in a building; Elsewhere in a building; Social media of my educational institution; Newsletter of my educational institution; Don't remember; Elsewhere, please describe: ___]
Frequency*	1 item, categorical	n/a	How often did you see the information? If you don't remember exactly, please estimate. For example: if you encountered the same information twice on a day, and other information on a different day, this counts as 3 times in total.<br>[1-2 times; 3-4 times; 5-6 times; 7-8 times; 8-9 times; more than 10 times]
Evaluation*	3 items; five-point semantic differential scales	n/a	How do you evaluate the information about prevention of virus spreading? The information was:<br>[Unclear – Clear; Unattractive – Attractive; Uncredible – Credible]
**Outcome measures (compliance and behavioral determinants)**	**Number of items and answer scales**	**Adapted from**	**Example item(s) and answer options**
Compliance with allocated	1 item, categorical	National Institute for	<u>In the past four weeks</u>, how often did you cough or sneeze in your <u>elbow</u>, instead of, for example, in your hand or in the air?

behavioral measure		Public Health and the Environment (RIVM) (2021)	[Never; Sometimes; Regularly; Frequently; Always; I didn't cough or sneeze]<br>*or*[†]<br>In the past four weeks (1 month), did you go outside when you were ill with respiratory infection symptoms (like coughing, sneezing, a sore throat, and a runny or stuffy nose)?<br>[No, I have not been ill with respiratory infection symptoms in the past four weeks; No, I have been ill with those symptoms but I did not go outside; Yes, only for urgent medical assistance; Yes, only after I did a Covid-19 (self) test and it was negative; Yes, only to get some fresh air or to walk my dog; Yes, I went outside for a different reason]
Intention to comply	2 items; 5-point semantic differential scale	Ajzen (2006)	In the coming four weeks (1 month), if you cough or sneeze, how often do you intend to cough or sneeze into your elbow?<br>*or*<br>In the next four weeks (1 month), when you are ill with respiratory infection symptoms, how often will you try to stay at home?<br>[Never – Always]
Attitude (affective and cognitive)	3 items; 5-point semantic differential scale	National Institute for Public Health and the Environment (RIVM) (2021);<br><br>Ajzen (2006)	I think coughing and sneezing into my elbow (instead of for example into my hand or in the air) is:<br>*or*<br>I think staying at home when I am ill with respiratory infection symptoms is:<br>[Not at all bothersome – Very bothersome; Bad – Good; Unwise – Wise]
Social norms (descriptive and injunctive)	4 items;<br>5-point Likert scales	National Institute for Public Health and the Environment (RIVM) (2021);	Most people in my direct surroundings cough and sneeze into their elbow.<br>*or*<br>Most people in my direct surroundings think it is important that I stay at home when I am ill with respiratory infection symptoms.<br>[Totally disagree – Totally agree]

		Ajzen (2006)	
Moral norm	2 items; 5-point Likert scales	Ajzen (1991), Beck & Ajzen (1991)	I feel guilty when I do not cough and sneeze in my elbow.<br>*or*<br>I feel responsible to stay at home when I am ill with respiratory infection symptoms.<br>[Totally disagree – Totally agree]
Self-efficacy	2 items; 5-point semantic differential scales	National Institute for Public Health and the Environment (RIVM) (2021); Ajzen (2006)	In the next four weeks (1 month), I think coughing or sneezing in my elbow is:<br>[Very difficult – Very easy]<br>*or*<br>In the next four weeks (1 month), if I want to, I am able to stay at home when I am ill with respiratory infection symptoms.<br>[Definitely not – Definitely yes]
Response efficacy	2 items; 5-point Likert scales	National Institute for Public Health and the Environment (RIVM) (2021); Andersson & Norberg (2023)	'Viruses' in this context means viruses that can cause respiratory infections, such as the flu or Covid-19.<br>Coughing and sneezing into my elbow helps to prevent the spreading of viruses.<br>*or*<br>Staying at home when you are ill with respiratory infection symptoms protects others from becoming infected with a virus.<br>[Totally disagree – Totally agree]
Knowledge of the allocated behavioral measure	3 items; categorical	n/a, scale developed based on potential misconceptions about the two risk-reducing behaviors	Coughing and sneezing in your elbow is only effective when you are not wearing a face mask.<br>*or*<br>Staying at home when you are ill with respiratory infection symptoms, helps to reduce the number of virus infections.<br>[True – Not true – Don't know]

Self-declared reasons not to comply with the allocated behavioral measure	1 item; open question	n/a	What would be reasons for you, not to cough and sneeze into your elbow as much as possible?<br>*or*<br>What would be reasons for you, not to stay at home as much as possible when you are ill with respiratory infection symptoms, like coughing, sneezing, a sore throat or a runny or stuffy nose?
**Personal characteristics**	**Number of items and answer scales**	**Adapted from**	**Example item(s) and answer options**
Risk perception (cognitive, i.e,. perceived severity and susceptibility, and affective, i.e., worry; regarding the self and others; targeting Covid-19 and the flu)	12 items; 5-point semantic differential scales	Hilverda & Vollmann (2021); National Institute for Public Health and the Environment (RIVM) (2021)	How likely do you think it is that you will become infected with the coronavirus in the coming month? It is about how big you think that this chance is. [very unlikely – very likely]; How severe would it be for you personally, if you became infected with the coronavirus? [not severe at all – very severe]; How worried are you, about becoming infected yourself with the coronavirus? [not worried at all – very worried]<br><br>Imagine that you would be infected with the flu. How likely do you think it is, that you will infect others? [very unlikely – very likely]; How bad would you feel to infect another person with the flu? [not bad at all – very bad]; How worried are you, that people who are important to you get infected with the flu? [not worried at all – very worried]
Knowledge of respiratory viruses	3 items; categorical	3 (out of 8) items addressing knowledge of risk-reducing behavior (Sattler et al., 2023)	Are the following statements true or not true? By 'viruses,' we mean viruses that can cause respiratory infections, such as the flu or Covid-19.<br>Antibiotics are effective against viruses. [True – False – Don’t know]
Trust in Dutch government regarding Covid-	3 items; 5-point Likert scales	National Institute for Public Health	During the Covid-19 crisis, I trusted that the Dutch government generally:<br>… Considered the right balance between various societal interests.<br>… Took the right measures to limit spreading of the virus.

19 crisis (competence; value similarity)		and the Environment (RIVM) (2021); Siegrist et al. (2003)	… Communicated openly and honestly about the purpose and efficiency of the various measures. [Totally disagree – Totally agree]
Prosocial orientation	2 items; 5-point semantic differential scale	n/a, scale based on 'self-other' categories used as experimental manipulation conditions in Jiang & Amponsah Dodoo (2021)	I think preventing the spreading of viruses that cause respiratory infections (such as the flu or Covid-19), is important to protect:<br>[Mainly myself – Mainly friends and family]<br>[Mainly myself – Mainly other people (not friends and family)]
Perceived health status	1 item; 5-point semantic differential scale	National Institute for Public Health and the Environment (RIVM) (2021)	How is your health in general? Generally, my health is: [Very bad – Very good]
Participation in T0*	1 item; categorical	n/a	Did you fill in a similar survey questionnaire about prevention of virus spreading a few weeks ago? [Yes; No]
**Anticipated intention following exposure to the three persuasive messages in a**	**Number of items and answer scales**	**Adapted from**	**Example item(s) and answer options**

**hypothetical high-risk pandemic scenario***			
Anticipated intention*	6 items; 5-point semantic differential scales	Items for “Intention to comply”	Currently, relatively few people suffer from severe illness caused by respiratory infections such as the flu and Covid-19. However, imagine that a new virus emerges that is highly contagious and causes severe illness. For example, a new strain of the coronavirus. In this situation, there will be a lot of pressure on the healthcare system and there will be behavioral measures such as physical distancing of 1.5 meters.<br>Imagine the described situation, in which a new virus emerges that is highly contagious and causes severe illness. You subsequently encounter the information displayed in the poster in the surroundings of your educational institution.<br><br>How often do you think you plan to cough and sneeze into your elbow, rather than into your hand or in the air? And how often do you think other people will cough and sneeze into their elbow?<br>I will cough and sneeze into my elbow: [Never – Always]<br>Other people will cough and sneeze into their elbow: [Never – Always]<br>*or*<br>How often do you think you plan to stay at home when you are ill with symptoms indicative of a respiratory infection, such as coughing, sneezing, a sore throat, and a runny or stuffy nose? And how often do you think other people will stay at home?<br>I will stay at home: [Never – Always]<br>Other people will stay at home: [Never – Always]
**Total**	**61 items**		

Note: *items indicated with an asterisk were only included in the post-intervention questionnaire (T1). †Participants were randomly allocated to answer questions about either coughing and sneezing into the elbow, or staying at home when ill with respiratory infection symptoms. Items and answer scales that were adapted to a particular behavior were intended to be as similar as possible across the two behaviors. In this overview, different items are provided as examples if a scale consisted of multiple items.

References

Ajzen, I. (1991). The theory of planned behavior. *Organizational Behavior and Human Decision Processes, 50*, 179-211. https://doi.org/10.1016/0749-5978(91)90020-T

Beck & Ajzen, (1991). Predicting dishonest actions using the theory of planned behavior. *Journal of Research in Personality, 25*, 285-301. https://doi.org/10.1016/0092-6566(91)90021-H

Ajzen, I. (2006). Constructing a theory of planned behavior questionnaire: Conceptual and methodological considerations. Retrieved on 04-04-2023, from: http://people.umass.edu/aizen/tpb.html

Andersson, E. M. & Norberg, M. (2023). Reasons for actions? Trust in protective behaviors and safeguarding measures in the early phase of the Covid-19 pandemic in Sweden. *Preventive Medicine Reports, 32*, 102133. https://doi.org/10.1016/j.pmedr.2023.102133

CBS. (2022). *Nieuwe indeling bevolking naar herkomst*. Retrieved 26 April 2023, from https://www.cbs.nl/nl-nl/longread/statistische-trends/2022/nieuwe-indeling-bevolking-naar-herkomst/4-de-nieuwe-indeling-naar-geboren-in-nederland-en-herkomstland

Hilverda, F. & Vollmann, M. (2021). The role of risk perception in students' COVID-19 vaccine uptake: A longitudinal study. *Vaccines, 10,* 22. https://doi.org/10.3390/vaccines10010022

Jiang, M. & Amponsah Dodoo, N. (2021). Promoting mask-wearing in COVID-19 brand communications: Effects of gain-loss frames, self- or other-interest appeals, and perceived risks. *Journal of Advertising*, 50, 271-279. https://doi.org/10.1080/00913367.2021.1925605

National Institute for Public Health and the Environment (RIVM) (2021). Corona gedragsonderzoek dataset [Dataset of Study on behavioural measures and well-being Corona]. Retrieved on 3 April 2023, from https://data.rivm.nl/meta/srv/dut/catalog.search#/metadata/3639da42-78b4 466c-a3eb-9e67809405d2

Sattler, S., Maskileyson, D., Racine, E., Davidov, E., & Escande, A. (2023). Stigmatization in the context of the COVID-19 pandemic: a survey experiment using attribution theory and the familiarity hypothesis. *BMC Public Health, 23*, 521. https://doi.org/10.1186/s12889-023-15234-5

Siegrist, M., Earle, T.C. and Gutscher, H. 2003. Test of trust and confidence model in the applied context of electromagnetic field (EMF) risks. *Risk Analysis, 23* 705–716. https://doi.org/10.1111/1539-6924.00349

Turner, M. M., Jang, Y., Wade, R., Heo, J. R., Ye, Q., Hembroff, L. A., & Lim, J. I. (2023). The effects of moral norms and anticipated guilt on COVID19 prevention behaviors. *Current Psychology, 24*, 1-13. https://doi.org/10.1007%2Fs12144-023-04477-5
